# Biallelic *TET2* mutations confer sensitivity to 5**′**-azacitidine in acute myeloid leukemia

**DOI:** 10.1172/jci.insight.150368

**Published:** 2023-01-24

**Authors:** Friedrich Stölzel, Sarah E. Fordham, Devi Nandana, Wei-Yu Lin, Helen Blair, Claire Elstob, Hayden L. Bell, Brigitte Mohr, Leo Ruhnke, Desiree Kunadt, Claudia Dill, Daniel Allsop, Rachel Piddock, Emmanouela-Niki Soura, Catherine Park, Mohd Fadly, Thahira Rahman, Abrar Alharbi, Manja Wobus, Heidi Altmann, Christoph Röllig, Lisa Wagenführ, Gail L. Jones, Tobias Menne, Graham H. Jackson, Helen J. Marr, Jude Fitzgibbon, Kenan Onel, Manja Meggendorfer, Amber Robinson, Zuzanna Bziuk, Emily Bowes, Olaf Heidenreich, Torsten Haferlach, Sara Villar, Beñat Ariceta, Rosa Ayala Diaz, Steven J. Altschuler, Lani F. Wu, Felipe Prosper, Pau Montesinos, Joaquin Martinez-Lopez, Martin Bornhäuser, James M. Allan

**Affiliations:** 1 Medical Clinic and Polyclinic I, University Hospital Dresden, Technical University of Dresden, Dresden, Germany.; 2 Translational and Clinical Research Institute, Newcastle University, Newcastle upon Tyne, United Kingdom.; 3 Department of Hematology, Freeman Hospital, Newcastle upon Tyne, United Kingdom.; 4 Barts Cancer Institute, Queen Mary University of London, London, United Kingdom.; 5 Icahn School of Medicine at Mount Sinai, New York, New York, USA.; 6 MLL Munich Leukemia Laboratory, Munich, Germany.; 7 Department of Hematology, Clínica Universidad de Navarra, Instituto de Investigación Sanitaria de Navarra (IdiSNA), Pamplona, Spain.; 8 Hematological Diseases Laboratory, CIMA LAB Diagnostics, University of Navarra, Navarra, Spain.; 9 IdiSNA, Navarra, Spain.; 10 Hematology Department, Hospital 12 de Octubre (i+12), Centro Nacional de Investigaciones Oncológicas (CNIO), Complutense University, Madrid, Spain.; 11 Department of Pharmaceutical Chemistry, School of Pharmacy, University of California, San Francisco, San Francisco, California, USA.; 12 Hospital Universitario y Politécnico La Fe, Valencia, Spain.; 13 National Center for Tumor Diseases, Dresden, Germany.

**Keywords:** Hematology, Cancer, Leukemias, Molecular genetics

## Abstract

Precision medicine can significantly improve outcomes for patients with cancer, but implementation requires comprehensive characterization of tumor cells to identify therapeutically exploitable vulnerabilities. Here, we describe somatic biallelic *TET2* mutations in an elderly patient with acute myeloid leukemia (AML) that was chemoresistant to anthracycline and cytarabine but acutely sensitive to 5*′*-azacitidine (5*′*-Aza) hypomethylating monotherapy, resulting in long-term morphological remission. Given the role of TET2 as a regulator of genomic methylation, we hypothesized that mutant *TET2* allele dosage affects response to 5*′*-Aza. Using an isogenic cell model system and an orthotopic mouse xenograft, we demonstrate that biallelic *TET2* mutations confer sensitivity to 5*′*-Aza compared with cells with monoallelic mutations. Our data argue in favor of using hypomethylating agents for chemoresistant disease or as first-line therapy in patients with biallelic *TET2*-mutated AML and demonstrate the importance of considering mutant allele dosage in the implementation of precision medicine for patients with cancer.

## Graphical abstract

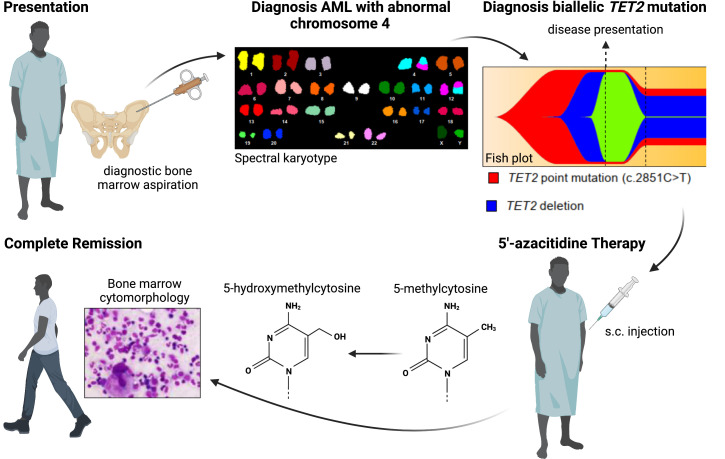

## Introduction

Acute myeloid leukemia (AML) is the exemplar of how interrogation of the somatic genome has facilitated understanding of disease pathogenesis and led to the development of novel therapies and stratified treatment approaches for some disease subgroups (1, 2). For example, the outcome of t(15;17)-positive acute promyelocytic leukemia has been revolutionized by the introduction of differentiation chemotherapy targeted against the promyelocytic leukemia–retinoic acid receptor–α fusion oncoprotein that defines this subgroup of AML (3, 4). Despite this success, therapeutic options for the majority of patients with AML are limited and outcome remains very poor, with a 5-year overall survival (OS) of just 15% (5). Subsequent refinement of the AML genomic landscape has revealed a plethora of somatically mutated genes, including *TET2*, which has been associated with poor outcome following treatment with standard anthracycline and nucleoside analog-based chemotherapy (1, 6).

Given the ineffectiveness of standard chemotherapy for many patients and the resulting poor outcome, it is essential to fully understand how somatic genetics can be utilized to identify vulnerabilities that can be therapeutically exploited using existing treatments and the expanding catalog of new agents. To this end, we present data that biallelic *TET2* mutations in AML confer sensitivity to hypomethylating chemotherapy. The TET2 enzyme catalyzes DNA demethylation by converting 5′-methylcytosine to 5′-hydroxymethylcytosine, and loss or attenuation of TET2 function leads to a somatically acquired global genomic hypermethylation and transcriptional and phenotypic reprogramming underpinning the development of a leukemia phenotype (7). As such, it is mechanistically plausible that hypomethylating chemotherapy could be particularly effective in TET2-null AML. These data serve as a paradigm for clinical diagnostics incorporating comprehensive genomic analyses to implement first-line therapies with a higher likelihood of response in patients with AML.

## Results

### AML index case with biallelic TET2 mutations.

**Figure 1:**
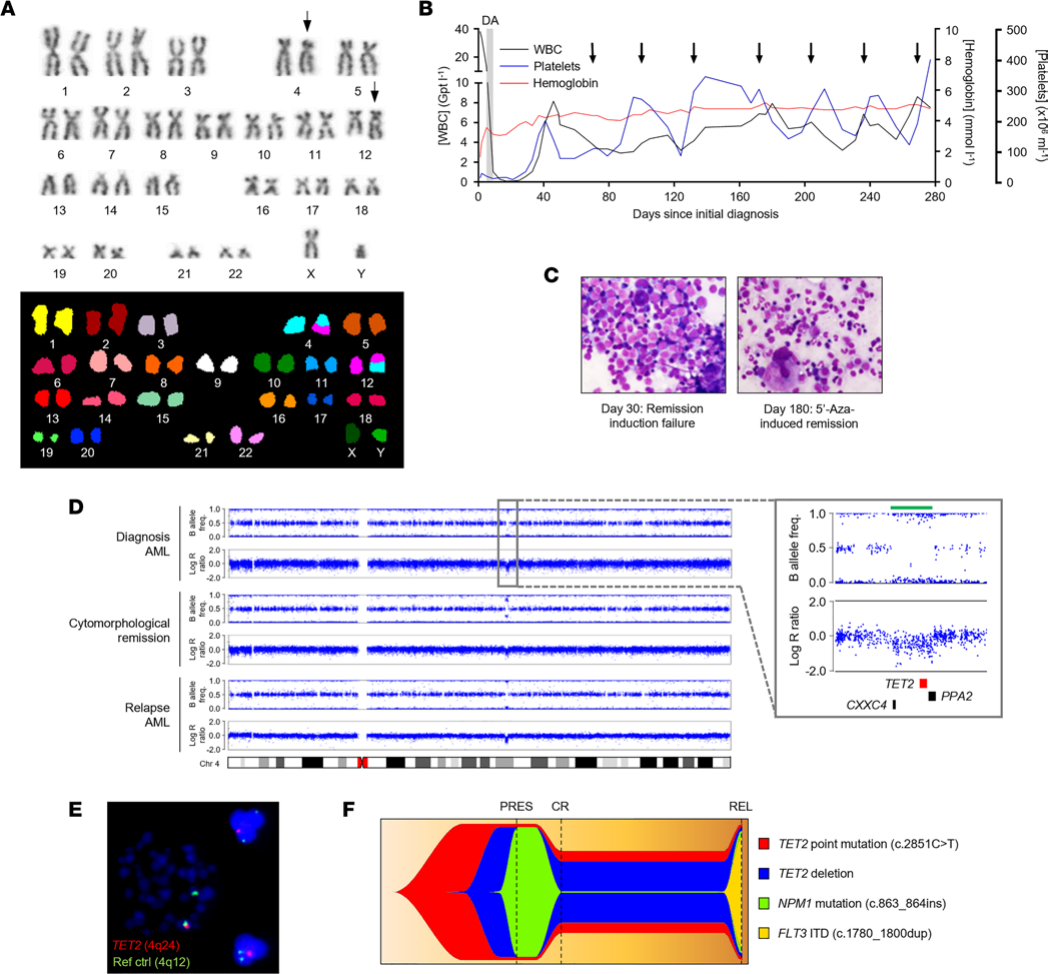
Biallelic *TET2* mutation in a patient with 5′-azacitidine–sensitive AML. (**A**) Identification of the t(4;12) translocation by G-banding (top) and spectral karyotyping (bottom) in leukemic blasts from the AML index patient (UPN01). Translocated chromosomes 4 and 12 are indicated with arrows. (**B**) Hemoglobin, platelet, and white blood cell (WBC) counts from diagnosis to relapse of UPN01. Gray shading indicates period of induction chemotherapy with daunorubicin and Ara-C (DA). Initiations of palliative 5′-Aza treatment cycles are indicated with arrows. Gpt, giga-particles. (**C**) Giemsa-stained BM smears at day 30 (left) (after failed induction chemotherapy) and day 180 (right) (during 5′-Aza–induced remission) in UPN01. Images are 500× original magnification. (**D**) High-density array copy number profiles of chromosome 4 from leukemic blasts of UPN01 at AML presentation, during CR, and at relapse. Points represent individual SNPs, aligned relative to their position on chromosome 4 (indicated by the ideogram). Copy number is measured as log R ratio, with 0 indicating diploid SNPs and positive and negative values indicating gain and loss, respectively. B allele frequency represents the ratio of the 2 alleles of each SNP such that 0.5 indicates allele heterozygosity and 0 and 1 indicate homozygosity. Inset shows expanded view of the boxed region. Green bar above plots highlights focal deletion within 4q24 encompassing *TET2*, *CXCC4*, and *PPA2* (locations indicated by bars below plots). (**E**) FISH on leukemic blasts from UPN01 showing *TET2* (shown in red) deletion in a metaphase cell (left of image) and 2 interphase cells (right of image). A probe binding within 4q12 (green) was used as reference. Original magnification, ×600. (**F**) Fish plot derived from sequencing analysis of leukemic blasts from UPN01 showing temporal acquisition of a *TET2* point mutation, a *TET2* deletion, an *NPM1* insertion mutation, and a *FLT3* internal tandem duplication (ITD). Dashed lines represent time points at which blasts were analyzed. 5′-Aza, 5′-azacitidine; PRES, disease presentation; CR, complete remission; REL, relapse.

We describe a 76-year-old man who presented with AML characterized by a t(4;12) translocation but no other cytogenetic abnormalities [46,XY,t(4;12) (q2?;q13)(12)/46,XY(10)] as identified by G-banding and confirmed by spectral karyotyping (Figure 1A). Standard 3+7 induction chemotherapy with daunorubicin and cytarabine (Ara-C) gave rise to a reduction in white blood cell count (Figure 1B). However, the patient became pancytopenic and developed acute septicemia requiring intensive care, intravenous antibiotics, and vasopressor therapy. Following recovery, blasts persisted in the bone marrow (BM) (29% at day 30; Figure 1C), indicating chemoresistant disease. The patient was subsequently treated with single-agent 5′-Aza monthly as palliation (Figure 1B), which unexpectedly resulted in prolonged complete morphological remission (CR) characterized by restoration of normal morphology (Figure 1C). The patient remained in CR for 24 months prior to emergence of relapsed AML. Relapsed disease was treated with subcutaneous Ara-C and sorafenib but was unresponsive to chemotherapy, and the patient died 28 months after first diagnosis. Autopsy revealed subtotal AML BM infiltration and multiple extramedullary AML sites, including lymph nodes and numerous parenchymatous organs (Supplemental Figure 1; supplemental material available online with this article; https://doi.org/10.1172/jci.insight.150368DS1).

To investigate the molecular basis underlying the prolonged response to 5′-Aza in this patient, we performed exome sequencing, interphase FISH, and single nucleotide polymorphism (SNP) array analysis of BM at AML presentation, during morphological remission, and at relapse. SNP array analysis demonstrated that the major cell clone at presentation was characterized by a focal 1.1 Mb deletion encompassing the *TET2*, *CXXC4*, and *PPA2* genes (Figure 1D). Interphase FISH on diagnostic BM demonstrated that approximately 95% of cells carried the *TET2* deletion (Figure 1E). Additionally, the retained *TET2* allele harbored a nonsense base substitution mutation in exon 3 affecting codon 939 (c.2815C>T, Q939*; Supplemental Figure 2), which was detected in 96% of cells. The presentation AML was also characterized by a heterozygous *NPM1* mutation (c.863_864insTCTG; Supplemental Figure 3) in all cells with *TET2* deletion. Integration of SNP array, exome, and FISH data was used to infer tumor phylogeny, which indicated that disease pathogenesis was initiated by the *TET2* nonsense mutation with subsequent deletion of the second *TET2* allele, followed by acquisition of the *NPM1* mutation (Figure 1F). Both the *TET2* gene deletion and the base substitution mutation were also present at high levels in the remission BM, despite this appearing morphologically normal (Figure 1, C and F, and Supplemental Figure 2). Although not discernible in the remission BM, the *NPM1* mutation was presumed to have persisted at levels below detection given that it was a prominent feature of relapsed disease, in addition to the *TET2* gene deletion and *TET2* base substitution (Figure 1F and Supplemental Figures 2 and 3). The relapse was also characterized by a heterozygous *FLT3* internal tandem duplication (c.1780_1800dupTTCAGAGAATATGAATATGAT; Supplemental Figure 4) in 80% of cells, which was not discernible in diagnostic or remission samples (Figure 1F and Supplemental Figure 4).

These data demonstrate that 5′-Aza treatment almost completely eliminated the *TET2/NPM1*-mutated clone dominant at disease presentation. Although also reduced by 5′-Aza treatment, ancestral AML cells carrying biallelic *TET2* mutations but negative for the *NPM1* mutation retained viability and presumably reacquired the ability to differentiate and recapitulate normal hematopoiesis, rendering a cytomorphological remission. Based on these observations, we hypothesized that mutant *TET2* allele dosage could affect cellular response and sensitivity to 5′-Aza.

### Biallelic TET2 mutations result in a hypermethylation phenotype in AML cells and confer sensitivity to 5′-Aza hypomethylating chemotherapy in vitro and in vivo.

**Figure 2:**
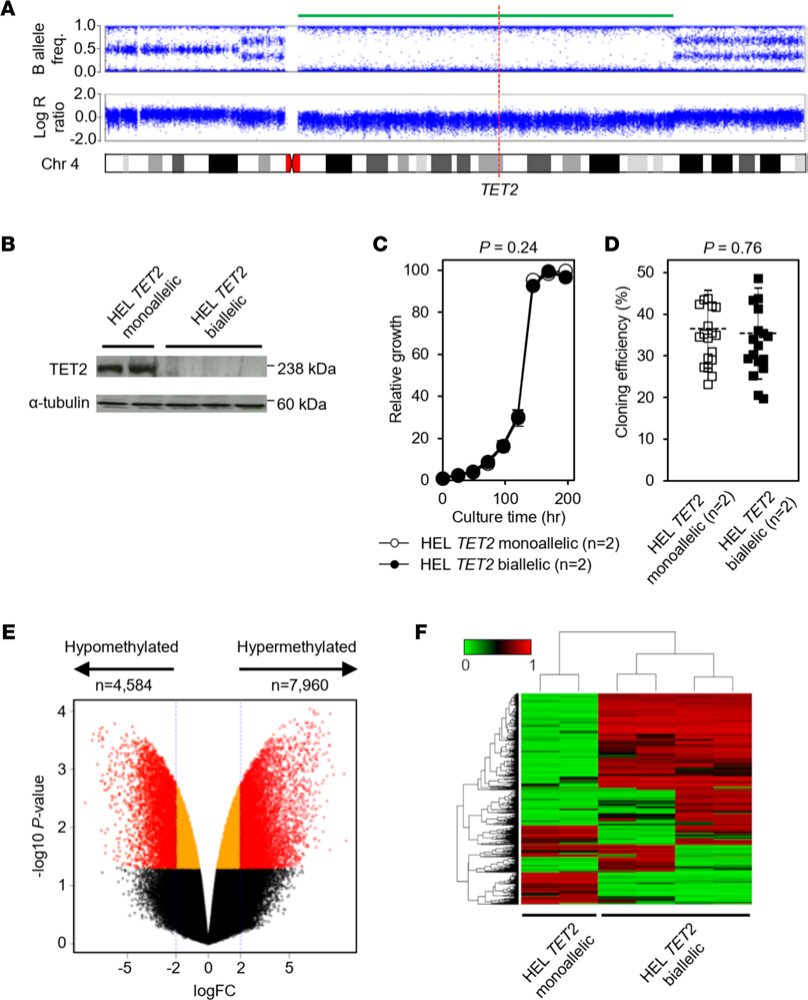
Complete loss of *TET2* expression confers a hypermethylation phenotype. (**A**) High-density array copy number profile of chromosome 4 from HEL cells showing large deletion (green bar) affecting the q arm, including *TET2* (position indicated by dashed red line). (**B**) Immunoblot showing TET2 protein expression in 2 representative parental *TET2* monoallelic HEL cell clones (HEL *TET2* monoallelic) and 3 representative *TET2* CRISPR/Cas9-mutated HEL cell clones (HEL *TET2* biallelic). α-Tubulin was used as loading control. (**C**) Growth kinetics in suspension culture of HEL *TET2* monoallelic (unfilled circles) and HEL *TET2* biallelic (filled circles) cell clones. Cells were seeded at low density, and growth (relative to initial density) was determined at regular intervals. Data represent mean and SD of indicated number of clones from 3 independent experiments. *P* value calculated by 1-way ANOVA. (**D**) CE was calculated for HEL *TET2* monoallelic (unfilled squares) and HEL *TET2* biallelic (filled squares) clones after 30 days culture in soft agar. Mean and SD of indicated number of clones from 7 independent experiments are shown. *P* value calculated by 2-tailed Student’s *t* test. (**E**) Volcano plot demonstrating differences in CpG methylation between HEL *TET2* monoallelic (*n* = 2) and HEL *TET2* biallelic (*n* = 4) clones. Plot was constructed using fold-change (log_2_FC) values, and adjusted *P* values and points represent individual CpG probes, colored such that significantly differentially methylated probes (*P* < 0.05 and |log_2_FC| ≥ 2) are in red. Orange points represent probes that reach significance (*P* < 0.05) but are not differentially methylated (|log_2_FC| < 2), and black points represent nonsignificant (*P* ≥ 0.05) probes. (**F**) Unsupervised hierarchical clustering of the top 1,500 differentially methylated CpG probes across all samples resulted in distinct clustering of parental HEL *TET2* monoallelic (*n* = 2) and HEL *TET2* biallelic (*n* = 4) cell clones. Rows in the heatmap represent CpG probes, and vertical columns represent cell clones. Color key indicates level of methylation at CpGs.

To test whether biallelic *TET2* mutations sensitize AML cells to 5′-Aza, we used CRISPR/Cas9 gene editing to completely inactivate *TET2* in the HEL AML cell line. HEL cells, derived from a 30-year-old male with erythroleukemia, have a complex hypotriploid karyotype with 60–64 chromosomes (Supplemental Table 1) and are reported to carry a monoallelic *TET2* gene deletion (8). Consistently, high-density SNP array analysis demonstrated that HEL cells carried a large deletion and concomitant loss of heterozygosity (LOH) affecting the majority of the long arm of chromosome 4, which includes the *TET2* gene (Figure 2A). Following transduction of HEL cells with CRISPR/Cas9 directed to *TET2*, Sanger sequencing revealed that the retained *TET2* allele was mutated in several independent clones. In particular, a 4 bp deletion in exon 6 (Supplemental Figure 5) was frequently observed. Regardless of the underlying mutation, HEL clones from independent CRISPR/Cas9 transductions were consistently null for TET2 protein expression (Figure 2B). Biallelic *TET2* mutations and consequent complete loss of TET2 protein expression did not affect proliferation kinetics of HEL clones in liquid media (Figure 2C) or cloning efficiency (CE) in soft agar (Figure 2D).

Analysis on human methylation (Infinium HumanMethylation450K, Illumina) arrays revealed the acquisition of an overall hypermethylation genotype in TET2-null HEL clones with biallelic *TET2* mutations (HEL *TET2* biallelic clones) compared with parental HEL clones with a monoallelic *TET2* deletion (referred to as HEL *TET2* monoallelic clones) (Figure 2E). Specifically, 7,960 of 410,811 probes (1.9%) were significantly hypermethylated (log_2_FC ≥ 2), and 4,584 probes (1.1%) were significantly hypomethylated (log_2_FC ≤ –2) in HEL *TET2* biallelic clones (*n* = 4) compared with HEL *TET2* monoallelic clones (*n* = 2) (Figure 2E and Supplemental Table 2). Differentially methylated probes mapped to loci throughout the genome (Supplemental Table 2). Unsupervised hierarchical clustering demonstrated clustering of HEL clones based on *TET2* mutation status (Figure 2F).

**Figure 3:**
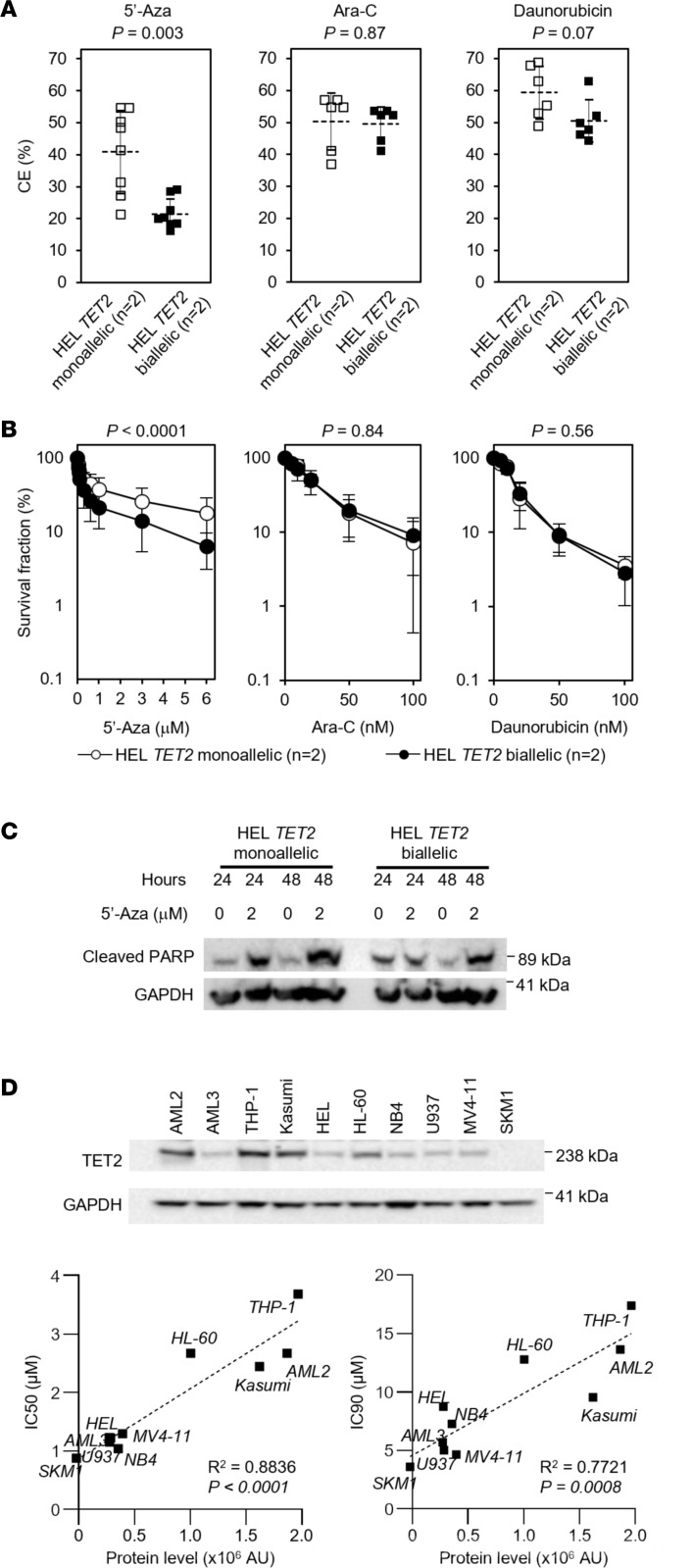
Cells with biallelic *TET2* mutations are sensitive to the hypomethylating agent 5′-Aza in in vitro model systems. (**A**) Parental *TET2* monoallelic HEL cell clones (HEL *TET2* monoallelic; unfilled symbols) and *TET2* CRISPR/Cas9-mutated HEL cell clones (HEL *TET2* biallelic; filled symbols) were cultured in soft agar supplemented with 1 μM 5′-Aza (left), 20 nM Ara-C (center), or 20 nM daunorubicin (right), and CE (relative to respective vehicle control–treated cells) was determined after 30 days. Mean and SD of indicated number of clones from 3 independent experiments shown. *P* values calculated by Student’s *t* test (2-tailed). (**B**) Parental *TET2* monoallelic HEL cell clones (HEL *TET2* monoallelic; unfilled symbols) and *TET2* CRISPR/Cas9-mutated HEL cell clones (HEL *TET2* biallelic; filled symbols) were treated with 5′-Aza (left), Ara-C (center), or daunorubicin (right), and cell density (relative to respective vehicle control–treated cells) was determined after 96 hours. Data represent mean and SD of indicated number of clones from 3 independent experiments. *P* values calculated by 2-way ANOVA. (**C**) Western blot showing cleaved PARP in HEL cells with monoallelic and biallelic *TET2* mutations following exposure to 2 mM 5′-Aza over 48 hours. GAPDH was used as a loading control. (**D**) Western blot (top) showing TET2 protein expression in a panel of 10 AML cell lines. GAPDH was used as a loading control. TET2 protein expression was quantified in each cell line and plotted against 5′-Aza IC_50_ (left) and IC_90_ (right) values. AU were measured by the Fuji LAS-300 Image Analyzer.

When treated with 5′-Aza in vitro, HEL *TET2* biallelic clones had significantly lower CE (*P* = 0.003) and proliferation in liquid culture (*P* < 0.001) compared with isogenic parental HEL *TET2* monoallelic clones (Figure 3, A and B). In contrast, *TET2* mutation load did not affect CE or cell proliferation following treatment with Ara-C or daunorubicin (Figure 3, A and B). *TET2* mutation status did not affect apoptosis induction in response to 5′-Aza, as measured by the induction of cleaved poly (ADP-ribose) polymerase (PARP) (Figure 3C) or the induction of cells with sub-G1 DNA content (Supplemental Figure 6), suggesting that complete loss of TET2 expression does not sensitize cells to 5′-Aza–induced apoptosis.

Having demonstrated that HEL cells null for TET2 protein expression were sensitive to growth inhibition by 5′-Aza, we sought to test the hypothesis in a second AML cell line mutant for *TET2*. SKM1 cells have a monoallelic *TET2* mutation (c.4253_4254insTT, p.1419fsX30) (9) but are phenotypically null for TET2 protein expression (Figure 3D), despite having an intact WT *TET2* allele. Nevertheless, consistent with the HEL cell data, TET2-null SKM1 cells are acutely sensitive to 5′-Aza (Figure 3D). We also quantified TET2 protein levels in a panel of 9 additional AML cell lines and determined sensitivity to 5′-Aza (Figure 3D). Strikingly, there was significant correlation between TET2 protein levels and 5′-Aza IC_90_ (*R*^2^ = 0.77, *P* = 0.0008) and IC_50_ (*R*^2^ = 0.88, *P* < 0.0001) (Figure 3D). THP-1 cells had the highest TET2 protein expression and were the most resistant to 5′-Aza. In contrast, SKM1 cells had the lowest TET2 protein expression and were the most sensitive to 5′-Aza.

**Figure 4:**
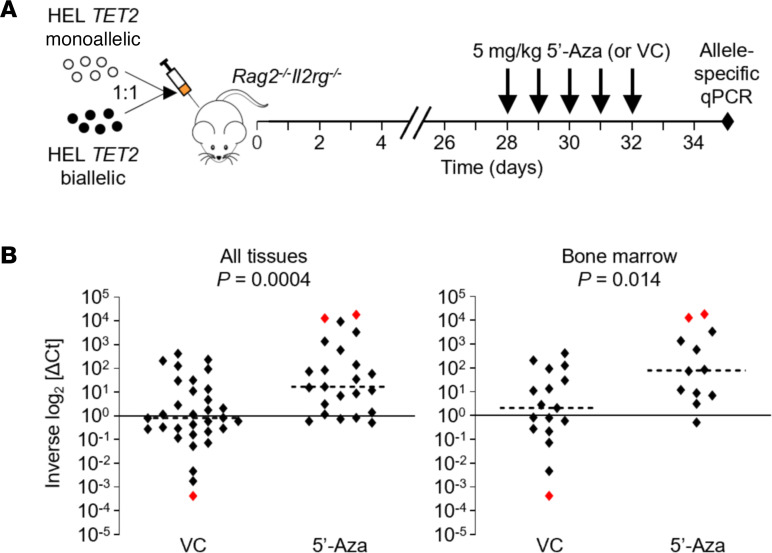
Cells with biallelic *TET2* mutations are subject to 5′-Aza–induced negative selection in an orthotopic AML mouse model. (**A**) Schematic of orthotopic AML mouse model. HEL *TET2* monoallelic and HEL *TET2* biallelic cell clones were coinjected in a 1:1 ratio into the femurs of *Rag2^−/−^Il2rg^−/−^* mice. Treatment with 5′-Aza (5 mg/kg daily for 5 days) or vehicle control (VC) was initiated on day 28 (postinjection), and animals were euthanized on day 35 for *TET2* allele-specific qPCR analysis of harvested tissues. (**B**) Tissue samples collected from mice were analyzed by custom *TET2* allele-specific qPCR assay. Shown are inverse log_2_ [ΔCt] values, which represent relative expression of the WT versus the 4 bp deleted *TET2* allele in individual samples and are the means of triplicate reactions. Inverse log_2_ [ΔCt] of 1 indicates a 1:1 ratio between the WT and 4 bp deleted *TET2* alleles (and hence HEL *TET2* monoallelic and HEL *TET2* biallelic clones), whereas inverse log_2_ [ΔCt] > 1 or inverse log_2_ [ΔCt] < 1 indicates dominance of the WT (HEL *TET2* monoallelic) or 4 bp deleted (HEL *TET2* biallelic) allele, respectively. Red points indicate samples that were dominated entirely by 1 cell clone. Horizontal dashed lines represent median inverse log_2_ [ΔCt] values across all samples from VC-treated or 5′-Aza–treated mice. The left panel shows data from all harvested tissues (BM, peripheral blood, spleen, and tumors), and the right panel shows data from BM only. *P* values comparing inverse log_2_ [ΔCt] values from VC-treated and 5′-Aza–treated mice were calculated using a Mann-Whitney test.

We next sought to determine whether biallelic *TET2* mutations sensitize AML cells to 5′-Aza in vivo in an orthotopic xenograft mouse model. We used a competitive engraftment approach in which HEL cell clones with monoallelic and biallelic *TET2* mutations were cotransplanted into mice, and an allele-specific quantitative PCR (qPCR) assay for the WT and CRISPR/Cas9-modified *TET2* alleles was subsequently utilized to determine preferential engraftment and/or elimination of either cell clone, with or without treatment with 5′-Aza. Specifically, following validation of the qPCR assay (Supplemental Figure 7), HEL *TET2* monoallelic and HEL *TET2* biallelic cell clones were coinjected intrafemorally (IF) in a 1:1 ratio into *Rag2^−/−^Il2rg^−/−^* (129 × BALB/c) mice (day 0; Figure 4A). Physical symptoms associated with the proliferation of AML cells (including weight loss, limited mobility, and growth of leg tumors in some animals) became apparent approximately 4 weeks after IF injection. On day 28 mice began once-daily treatment with 5 mg/kg 5′-Aza (or VC) for 5 days and were then euthanized 3 days later (day 35 after engraftment and day 8 after the initiation of treatment) for sample collection and qPCR analysis (Figure 4A). Tissue samples were collected from 6 mice treated with 5′-Aza and 9 VC-treated mice. We were able to consistently amplify human *TET2* DNA in tissue obtained from injected femurs, as well as noninjected femurs, spleens, and other organs showing evidence of AML infiltration, yielding 55 individual samples (32 from VC-treated mice and 23 from 5′-Aza–treated mice). There was no overall mean preferential amplification of either the intact WT or CRISPR-modified *TET2* allele in all 32 tissue samples from VC-treated mice (median inverse log_2_ [ΔCt] = 0.81; Figure 4B), though the CRISPR-modified *TET2* allele slightly dominated in spleen samples (*P* = 0.047; Supplemental Figure 8), suggesting preferential engraftment of TET2-null cells specifically in this tissue. Conversely, in 5′-Aza–treated mice, the WT *TET2* allele was dominant in 19 of 23 (83%) tissue samples (median inverse log_2_ [ΔCt] = 16.81; Figure 4B), demonstrating significant negative selection against TET2-null cells (*P* = 0.0004) as a result of 5′-Aza treatment. There was also significant negative selection of TET2-null cells specifically in the BM of 5′-Aza–treated mice (median inverse log_2_ [ΔCt] = 2.09 and 79.34 for VC-treated and 5′-Aza–treated mice, respectively; *P* = 0.014), with 2 (of a total of 12 femurs from 5′-Aza–treated mice) completely negative for the CRISPR-modified *TET2* allele (Figure 4B).

Taken together, these data demonstrate that TET2-null cells are sensitive to the hypomethylating agent 5′-Aza both in vitro and in vivo, consistent with the response to 5′-Aza observed in the index AML case.

We next investigated whether knockdown or knockout of TET2 affects sensitivity to 5′-Aza in AML cell lines derived from primary AML that was not *TET2* mutant. shRNA-mediated knockdown of TET2 in THP-1 AML cells (10, 11) conferred sensitivity to the growth-inhibitory effects of 5′-Aza (*P* = 0.005), though the phenotype was relatively weak (Supplemental Figure 9). In contrast, CRISPR-mediated knockout of *TET2* in KG-1 AML cells (11) conferred resistance to the growth-inhibitory effects of 5′-Aza (*P* < 0.0001; Supplemental Figure 9). It should be noted that neither of these cell models were null for TET2, and both retained some residual protein expression (Supplemental Figure 9), which is possibly due to incomplete shRNA-mediated knockdown in THP-1 cells and incomplete CRISPR targeting in KG-1 cell populations, respectively.

### Gene expression analysis identifies downregulation of small nuclear ribonucleoprotein complex components and ATP binding cassette subfamily B member 1 drug efflux in cells with biallelic TET2 mutations.

**Figure 5:**
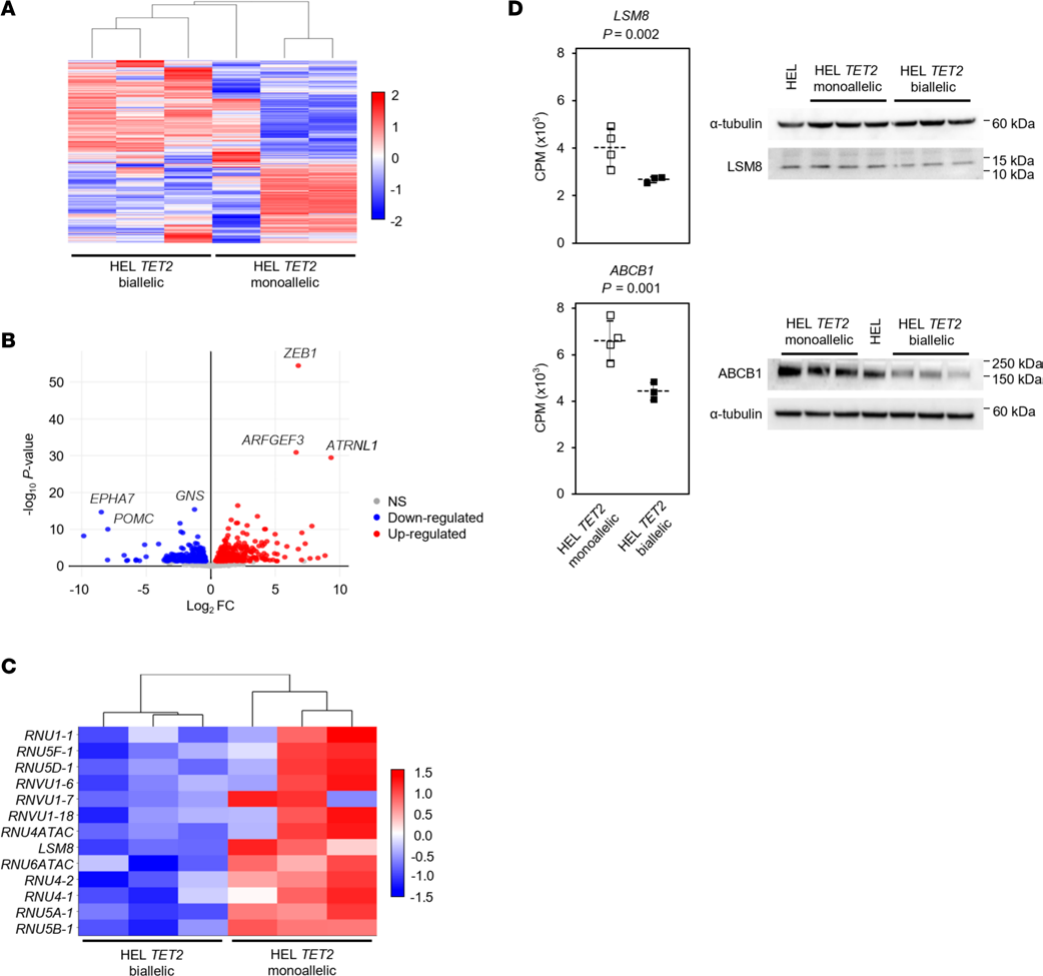
Differential gene expression in AML cells with monoallelic and biallelic *TET2* mutations. (**A**) Heatmap showing the top 1,500 differentially expressed transcripts in parental *TET2* monoallelic HEL cell clones (HEL *TET2* monoallelic; *n* = 3) and *TET2* CRISPR/Cas9-mutated HEL cell clones (HEL *TET2* biallelic; *n* = 3). Horizontal rows represent individual transcripts and each vertical column represents a cell clone. Color indicates relative expression, with downregulated and upregulated transcripts indicated in blue and red, respectively. (**B**) Volcano plot demonstrating significant differential gene expression (*P* < 0.05 and |log_2_FC| ≥ 0.3) in *TET2* CRISPR/Cas9-mutated HEL cell clones (HEL *TET2* biallelic; *n* = 3) relative to parental HEL clones with monoallelic *TET2* mutation (HEL *TET2* monoallelic; *n* = 3). Plot was constructed using log_2_FC values and adjusted *P* values, and points represent individual gene transcripts. Shown are 326 significantly downregulated transcripts (blue) and 369 significantly upregulated transcripts (red). Nonsignificant transcripts (*P* ≥ 0.05) are represented by gray points. Genes with particularly significant differential expression are labeled. (**C**) Heatmap showing differential expression of components of the spliceosomal snRNP complex (GO:009752) in parental *TET2* monoallelic HEL cell clones (HEL *TET2* monoallelic; *n* = 3) and *TET2* CRISPR/Cas9-mutated HEL cell clones (HEL *TET2* biallelic; *n* = 3). Horizontal rows represent genes and each vertical column represents a cell clone. Color indicates relative expression, as in **A**. (**D**) Transcript expression (expressed as counts per million [CPM] reads) of *LSM8* (top) and *ABCB1* (bottom) in parental *TET2* monoallelic HEL cell clones (HEL *TET2* monoallelic; unfilled bars) and *TET2* CRISPR/Cas9-mutated HEL cell clones (HEL *TET2* biallelic; filled bars). Data represent the mean and SD of indicated number of clones. *P* values are from differential expression analysis. Western blots to the right of the charts show corresponding protein expression in the individual cell clones included in RNA-sequencing analysis. α-Tubulin was used as a loading control.

RNA-sequencing analysis was performed to identify differentially expressed genes and potential mechanisms responsible for 5′-Aza sensitivity in HEL *TET2* biallelic cell clones. Upon unsupervised hierarchical clustering of transcript data, HEL cell clones clustered broadly by *TET2* genotype (Figure 5A), suggesting that complete loss of TET2 protein significantly affected transcription. Differential expression analysis identified 695 significantly differentially expressed transcripts (adjusted *P* [*P*_adj_] < 0.05; |log_2_FC| ≥ 0.3) in HEL *TET2* biallelic clones compared with HEL *TET2* monoallelic clones (Figure 5B and Supplemental Table 3). Gene Ontology analysis identified several significantly affected cellular components (Supplemental Table 4), of which the spliceosomal small nuclear ribonucleoprotein (snRNP) complex (GO:0097525) was the most significantly affected (*P*_adj_ = 8.7 × 10^–4^). In differential expressional analysis, the 12 RNA genes and 1 protein-coding gene (*LSM8*) that make up this complex were all significantly downregulated in TET2-null cells (Figure 5C and Supplemental Table 5). Likewise, spliceosomal tri-snRNP complex assembly (GO:0000244) was identified as the most significantly affected biological process (*P*_adj_ = 3.4 × 10^–4^; Supplemental Table 6). Downregulation of protein expression in TET2-null cells was validated for LSM8, consistent with transcript expression data (Figure 5D). Significant differences in expression were also identified for other genes that could affect cellular response to 5′-Aza (Supplemental Table 3). These included *ABCB1* (*MDR1*) (12), encoding a member of the ATP binding cassette (ABC) family of drug transporters, which was downregulated in TET2-null cells at the transcript (*P*_adj_ = 0.001) and protein level (Figure 5D).

**Figure 6:**
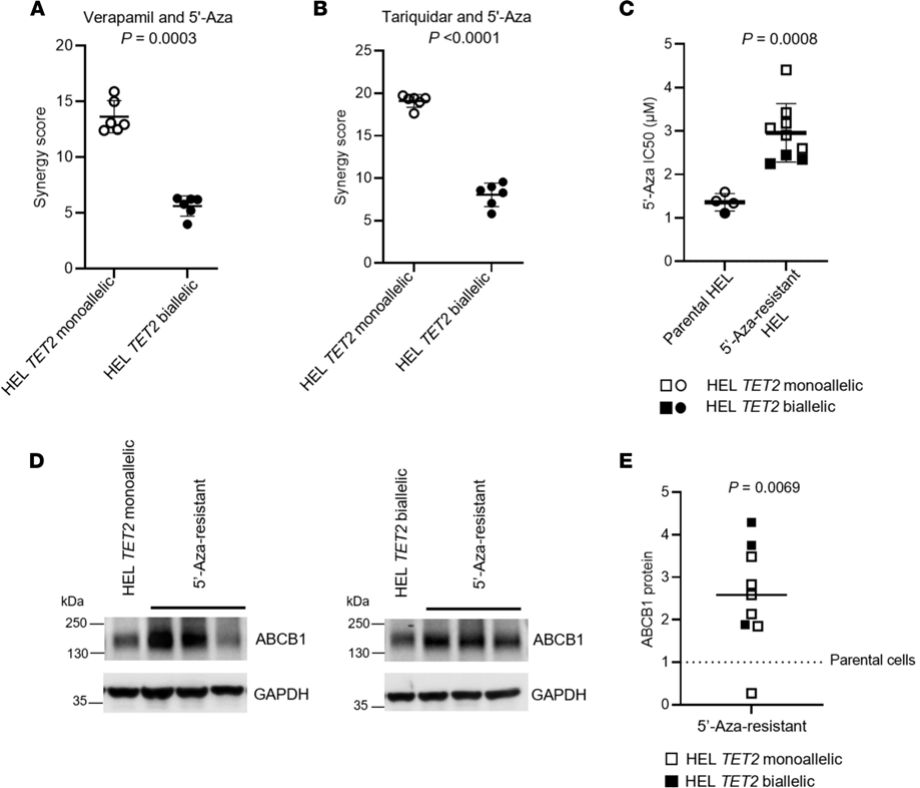
ABCB1 affects sensitivity to 5′-Aza in HEL AML cells. (**A**) Mean synergy scores for verapamil and 5′-Aza combination and for (**B**) tariquidar and 5′-Aza in combination, stratified by *TET2* mutant allele dosage. Synergy scores for verapamil/5′-Aza and tariquidar/5′-Aza were significantly higher for *TET2* monoallelic mutant HEL cell clones compared with *TET2* biallelic HEL cell clones (*P* = 0.0003 and *P* < 0.0001, respectively, paired 2-tailed Student’s *t* test). Data are derived from 6 independent experimental replicates using 2 independent cell clones for each *TET2* genotype. Data represent the mean and SD of indicated number of clones. (**C**) HEL AML cell clones were exposed to escalating doses of 5′-Aza to generate significantly resistant subclones (*P* = 0.0008, unpaired 2-tailed Student’s *t* test). Data show IC_50_ values for 5′-Aza–resistant subclones derived from parental cells with either *TET2* monoallelic mutation (unfilled symbols) or *TET2* biallelic mutation (filled symbols). Data represent the mean and SD of indicated number of clones. (**D**) Western blots show ABCB1 protein levels in 3 representative 5′-Aza–resistant derivatives from cells with either *TET2* monoallelic mutation (left) or *TET2* biallelic mutation (right). (**E**) ABCB1 protein levels were quantified in parental HEL cells, and 9 independent 5′-Aza–resistant derivatives with either *TET2* monoallelic mutation (unfilled symbols) or *TET2* biallelic mutation (filled symbols). ABCB1 protein levels were quantified and normalized to GAPDH, with the expression in each parental cell given a nominal value of 1. ABCB1 protein levels of 5′-Aza–resistant derivatives were significantly higher than their respective parental cells (*P* = 0.0069, paired 2-tailed Student’s *t* test). Solid horizontal line represents the median fold change in ABCB1 protein expression in 5′-Aza–resistant subclones relative to their respective parental cells (represented by the dashed horizontal line).

To further investigate the role of ABCB1 as a determinant of sensitivity to 5′-Aza, HEL AML cell clones were treated with ABCB1 inhibitors verapamil or tariquidar. When used in combination with 5′-Aza, both agents sensitized HEL AML cells to the growth-inhibitory effects of 5′-Aza. Moreover, cotreatment with either ABCB1 inhibitor and 5′-Aza was synergistic in HEL cell clones with monoallelic *TET2* mutation, which express high levels of ABCB1, but not in HEL cell clones with biallelic *TET2* mutation, which express low levels of ABCB1 (Figure 6, A and B, and Supplemental Figures 10 and 11). We next cloned HEL AML cells in soft agar supplemented with 10 μM 5′-Aza. Colonies that survived 5′-Aza exposure were all expanded and shown to be resistant to 5′-Aza (Figure 6C). Regardless of *TET2* mutant allele dosage, 8 of 9 HEL cell clones that were 5′-Aza resistant had upregulated ABCB1 protein expression relative to their respective parental cells from which they were derived (*P* = 0.0069, Figure 6, D and E). Taken together, these data demonstrate a role for ABCB1 as a determinant of sensitivity to 5′-Aza.

### Biallelic TET2 alterations in AML patients with cytogenetically discernible chromosome 4 aberrations.

The *TET2* locus can be somatically affected via numerous mechanisms, including point mutations as well as gains and losses of material, though how these give rise to biallelic *TET2* mutations remains unclear. To investigate, we screened the Study Alliance Leukemia (SAL) biobank for patients with AML presenting with a cytogenetically discernible aberration affecting chromosome 4, a population likely to be enriched for structural *TET2* alterations. However, because *TET2* base substitutions are reported with high frequency in cytogenetically normal AML (1), our approach of selecting cases with chromosome 4 abnormalities does not inform on the overall frequency of alterations in AML. A total of 30 cases recruited to the SAL biobank had a chromosome 4 aberration visible cytogenetically and had sufficient material for SNP array analysis and sequencing (Supplemental Table 7).

**Figure 7:**
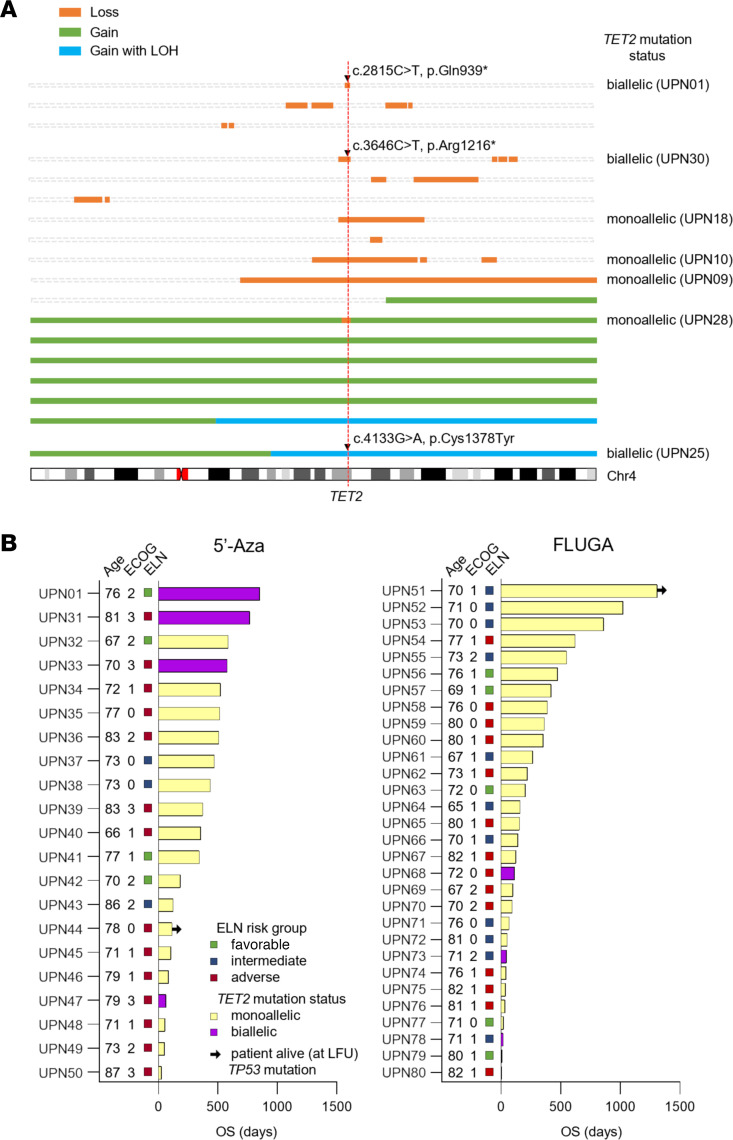
Somatic mutations affecting the *TET2* locus in AML patients with cytogenetic abnormality of chromosome 4 and response to treatment in AML patients with *TET2* mutation. (**A**) Illustrated are regions of copy number gain (green), gain with concomitant LOH (blue), and loss (orange) affecting chromosome 4 (discerned using high-density SNP array) in 18 AML patients with cytogenetically detectable abnormalities of chromosome 4. Base substitution mutations (indicated by black triangles) were determined by *TET2* exon sequencing. The vertical dashed red line indicates the location of the *TET2* gene. The mutation statuses of the 7 patients with loss-of-function *TET2* mutations are indicated to the right. Patient ID numbers are shown in parentheses for these patients. (**B**) Swimmer plots showing patients with *TET2*-mutated AML treated with either 5′-Aza (left) or low-dose Ara-C plus fludarabine (FLUGA) (right). The AML index case (UPN01) is included in the 5′-Aza swimmer plot for reference. Patients with biallelic *TET2* mutation (UPN01, UPN31, UPN33, UPN47, UPN68, UPN73, and UPN78) are represented by purple bars. All other patients had monoallelic *TET2* mutation discerned by whole-exome sequencing and are represented by pale yellow bars. European LeukemiaNet (ELN) favorable-, intermediate-, and adverse-risk groups are represented by green, blue, and red squares, respectively. ECOG, Eastern Cooperative Oncology Group performance score.

Gains affecting *TET2* were discernible by SNP array in 6 patients (all with trisomy 4 visible cytogenetically), which included 2 cases with homozygosity affecting most of the long arm of chromosome 4 (Figure 7A). One of these 2 patients (UPN25) also had a *TET2* base substitution (c.4133G>A, p.Cys1378Tyr) carried by almost 100% of the cells (Figure 7A and Supplemental Figure 12) and as such had biallelic mutations affecting *TET2*. Six cases had loss of genetic material affecting *TET2* discernible from SNP array data, which included 3 cases with large deletions (UPN09, UPN10, and UPN18) and 2 cases with a focal deletion in 1 allele and a nonsense mutation in the other allele (index case UPN01 and UPN30) (Figure 7A and Supplemental Figure 12). The sixth case (UPN28) had trisomy 4 but with a focal 585 kb deletion affecting the entire *TET2* gene, resulting in copy number reduction (<2 copies) and LOH (Figure 7A and Supplemental Figure 12). A further 6 cases had copy number alterations (5 with loss, 1 with gain) on chromosome 4, which did not affect the *TET2* locus (Figure 7A), and the remaining 12 cases had no evidence of *TET2* base substitution or gain or loss of material on chromosome 4. Although 2 of these 12 cases had trisomy 4 (UPN26 and UPN29) and 1 case had monosomy 4 (UPN13) visible cytogenetically, these aneuploidies were present in a minor subclonal population (Supplemental Table 7), which explains why they were not visible in the SNP array data. These data demonstrate that *TET2* alterations are complex, often involving gains or losses of material in combination with base substitution mutations.

### Biallelic TET2 alterations in patients with AML treated with 5′-Aza.

Having described a single patient with biallelic *TET2* mutation (index case UPN01), who responded very well to 5′-Aza, we sought to determine whether biallelic *TET2* mutation was also associated with a favorable response in other patients treated with 5′-Aza. Patients with AML over the age of 65 years were recruited to the para el Estudio de la Terapeutica en Hemopatias Malignas (PETHEMA-FLUGAZA) phase III clinical trial and were randomized to receive either 5′-Aza or low-dose Ara-C plus fludarabine (FLUGA) (13). A total of 50 patients had a *TET2* mutation identified by targeted sequencing, which included 6 patients with a mutant allele frequency more than 85%, indicative of biallelic *TET2* mutation (3 patients were randomized to each arm of the trial) (Supplemental Table 8). None of the 3 patients with biallelic *TET2* mutation randomized to the FLUGA arm (UPN68, UPN73, UPN78) achieved CR, and all had relatively short OS (111, 45, and 17 days) (Figure 7B and Supplemental Table 8). In contrast, 2 of the 3 patients with biallelic *TET2* mutation randomized to the 5′-Aza arm achieved CR (UPN31 and UPN33) and had prolonged OS (767 and 579 days) (Figure 7B and Supplemental Table 8). The third patient treated with 5′-Aza (UPN47) did not achieve CR and died (day 62) after cycle 1 with progressive disease (Figure 7B and Supplemental Table 8). Furthermore, all 3 patients with biallelic *TET2* mutation treated with 5′-Aza had ELN adverse-risk AML and an ECOG performance score of 3. Also, 1 of the patients with biallelic *TET2* mutation who responded to 5′-Aza (UPN33) had AML that was *TP53* mutant (Figure 7B and Supplemental Table 8).

In summary, we have identified 3 patients with biallelic *TET2* mutations who had a favorable response to single-agent 5′-Aza, including the index case (UPN01), who had disease resistant to standard daunorubicin and Ara-C remission induction chemotherapy.

## Discussion

Up to 30% of patients with AML present with a somatically acquired *TET2* mutation (14–20), with biallelic mutations representing a minority of all *TET2*-mutated AML cases (20–22). The prognostic effect of *TET2* mutation in AML treated with anthracycline and nucleoside analog-based regimens remains controversial (16, 18, 19), although meta-analyses suggest an association with poor prognosis (23, 24). As such, there is an urgent clinical need to identify novel therapeutic approaches to improve the outcome of *TET2*-mutated AML. Some studies have reported an association between *TET2* mutation and favorable outcome of myelodysplastic syndrome (MDS) following treatment with hypomethylating chemotherapy, such as 5′-Aza (25–29), though other studies have not replicated these findings (30). Our data demonstrate that single-agent 5′-Aza treatment of AML harboring biallelic *TET2* mutations can give rise to long-term CR, including disease otherwise refractory to standard 3+7 induction chemotherapy with daunorubicin and Ara-C and in patients with adverse-risk disease or poor performance status. Furthermore, using an isogenic model system, we demonstrate that biallelic *TET2* mutations confer cellular hypersensitivity to 5′-Aza in vitro, as well as significant negative selection when competitively xenografted with monoallelic *TET2*-mutated cells into the BM of mice (Figure 4).

It should be noted that our data were primarily generated using HEL AML cells, which were derived from primary AML with monoallelic *TET2* mutation. We also observed acute sensitivity to 5′-Aza in TET2-null SKM1 cells, which were also derived from *TET2*-mutant primary AML. We also investigated 5′-Aza sensitivity in AML cell lines not derived from TET2-mutant disease, and although we observed sensitivity in THP-1 cells with TET2 knockdown, we did not see the same phenotype in KG-1 cells with TET2 knockout, which were relatively resistant to 5′-Aza compared with *TET2* WT KG-1 cells. These data suggest that complete loss of TET2 expression might be required for sensitivity to 5′-Aza or that sensitivity is modified by other somatic mutations. For example, HEL and SKM1 cells are WT for *TP53* whereas THP-1 and KG-1 are both mutant for *TP53*, which is associated with poor outcome in AML (31, 32). Although we report 1 patient with *TET2* biallelic mutant, *TP53*-mutant AML who responded well to 5′-Aza (UPN33), all other patients with biallelic *TET2* mutations were WT for *TP53*. As such, further work is warranted to understand the impact of *TP53* and other somatic mutations in determining response to 5′-Aza in TET2-null AML.

An effect of mutant *TET2* gene dosage on response to therapy is perhaps not surprising given the evidence demonstrating that mutant allele dose also affects disease development. Specifically, monoallelic (*Tet2*^+/–^) and biallelic *Tet2* deletions (*Tet2*^–/–^) both result in myeloid malignancy in animal models, but the latency and OS are significantly shorter in *Tet2*-null (*Tet2*^–/–^) animals (33, 34). Furthermore, *Tet2*^–/–^ mice with myeloid disease also have more pronounced splenomegaly compared with heterozygous (*Tet2*^+/–^) littermates (33), and splenomegaly (and extramedullary disease) is a general feature of myeloid disease developing in *Tet2*-knockout mouse models (10, 34). Consistent with this, young, healthy mice null for *Tet2* have elevated extramedullary hematopoiesis in the spleen, which develops into splenomegaly concomitant with the onset of myeloid dysplasia (35). These observations are consistent with our data demonstrating a significant competitive advantage of TET2-null human cells to populate the spleen of engrafted animals and also that the index patient (UPN01) reported herein presented with splenomegaly and extramedullary disease. Taken together, these data suggest that TET2 loss could predispose to myeloid disease characterized by splenomegaly and extramedullary disease in general, which is mutant *TET2* gene dosage dependent, although investigation in large patient cohorts is warranted.

Our data suggest that complete loss of TET2 renders cells more sensitive to the antiproliferative effects of 5′-Aza, rather than enhancing susceptibility to drug-induced apoptosis, consistent with the observed negative selection against cells with biallelic *TET2* mutation observed in vivo. Despite this, 5′-Aza treatment rarely resulted in the complete elimination of TET2-null cells in mice, consistent with data from the index patient, in whom 5′-Aza–induced morphological remission was characterized by the persistence of cells with biallelic *TET2* mutations. Mutation persistence in morphological remission has been reported for several leukemia driver genes, including those characteristic of age-associated clonal hematopoiesis such as *TET2*, *DNMT3A*, *SRSF2*, *RUNX1*, and *ASXL1* (36–39). Likewise, persistence of *Tet2*-mutated cells has also been reported in animal models treated with 5′-Aza (40). Targeting 2 epigenetic layers in monoallelic *TET2*-mutated AML with 5′-Aza and lysine demethylase 1A inhibition has been demonstrated to be effective in primary AML cells ex vivo (41). However, a model analyzing responsiveness of biallelic *TET2*-mutated AML to 5′-Aza has not been reported to our knowledge.

Our data demonstrate that cells with monoallelic and biallelic *TET2* mutations have significantly different genomic methylation profiles (Figure 2). Although we observed a genome-wide shift toward hypermethylation in cells with biallelic *TET2* mutation, the effect was relatively modest, with large numbers of CpG sites that became hypomethylated. Consistent with this, we also noted upregulated transcript levels for numerous genes. As such, it seems unlikely that global genomic DNA methylation and concomitant global loss of expression are responsible for the observed sensitivity to 5′-Aza. Rather, the prevailing evidence suggests that the underlying mechanism conferring sensitivity to 5′-Aza is gene/pathway specific, and our investigations identified significant downregulation of spliceosomal snRNP complex components in cells with biallelic *TET2* mutations (Figure 5). The snRNP pathway has previously been implicated as a determinant of cellular sensitivity to 5′-Aza (42), though the underlying mechanisms remain to be fully deciphered. We also show that ABCB1 was downregulated in AML cells with biallelic *TET2* mutations and that inhibition of this efflux transporter sensitized to the growth-inhibitory effects of 5′-Aza. Inhibition of ABCB1 leads to increased intracellular accumulation of 5′-Aza in SKM1 AML cells (12, 43), providing further evidence that ABCB1 is involved in 5′-Aza efflux. Consistent with our data, treatment with a combination of 5′-Aza and erlotinib, which antagonizes ABCB1, is synergistically cytotoxic in several AML cell lines, including SKM1, MOLM-13, HL-60, and MV4-11 (12). We also demonstrate significant upregulation of ABCB1 protein expression in 5′-Aza–resistant HEL cell clones (Figure 6, D and E). Messingerova and colleagues (44) also reported upregulation of ABCB1 protein in 5′-Aza–resistant clones developed from SKM1 and MOLM-13 AML cell lines.

Mutations in other genes operating in the TET2 hydroxymethylation pathway are reported in AML, including *IDH1*, *IDH2*, and *WT1*. Mutations in *IDH1* and *IDH2* inhibit TET2 (and TET1 and TET3) function via production of 2-hydroxyglutarate (45). *WT1* mutations in AML (46) drive leukemogenesis via inhibition of TET2. As such, loss of WT1, IDH1, or IDH2 partially phenocopies loss of TET2 function and could sensitize AML cells to the inhibitory effects of 5′-Aza. In support of this notion, 5′-Aza as a single agent and particularly in combination with either ivosidenib (IDH inhibitor) or venetoclax (BCL2 apoptosis regulator inhibitor) has efficacy in *IDH*-mutated AML (47, 48). It will, therefore, be important to determine whether mutations in other members of the hydroxymethylation pathway confer sensitivity to 5′-Aza in AML, as we report here for biallelic *TET2* mutations. *TET2* mutations have also been reported in up to 28% of MDS and myeloproliferative neoplasms (15, 20, 49) and up to 50% of angioimmunoblastic T cell lymphoma (AITL), where they are associated with poor response to anthracycline-based chemotherapy (50). However, there is evidence of sensitivity to 5′-Aza in *TET2*-mutated AITL cases (51), with prolonged CR reported in 1 case with double (presumed biallelic) mutation (50).

Models for reliably predicting response to 5′-Aza in AML would be of clinical benefit. Our study suggests that *TET2* mutational profiling or TET2 protein expression analysis could potentially identify a subgroup of patients with disease that is null for protein expression and acutely sensitive to hypomethylating therapy, suggesting an alternative first-line therapy for frail AML patients or salvage therapy for patients with chemoresistant disease. There is potential value in advocating *TET2* mutational or protein expression profiling in elderly patients with AML, where disease is more likely to have evolved from *TET2* clonal hematopoiesis and therefore likely to be enriched for AML with biallelic *TET2* mutations and null for expression (20). Indeed, clinical studies in elderly patients with AML have already documented excellent responses to 5′-Aza in some patients (52), though the impact of TET2 status would need to be confirmed in prospective studies in all age groups. Likewise, we make a case for implementing *TET2* mutational or expression profiling in AML patients with extramedullary disease, particularly with splenomegaly, given our data linking biallelic *TET2* mutation with colonization of the spleen along with data from mouse models showing a proclivity of *Tet2* mutation to drive extramedullary hematopoiesis and myeloid disease.

In summary, the prevailing evidence argues in favor of investigating mutant *TET2* allele dosage and TET2 protein expression as determinants of sensitivity to 5′-Aza in large prospective studies of AML and other hematological conditions characterized by TET2 loss of function. However, comprehensive *TET2* mutational profiling that includes both sequence and copy number analysis would be required to identify patients with potentially complex alterations affecting the *TET2* locus. Furthermore, TET2 expression profiling could identify patients with disease that is null or low for protein expression regardless of gene mutation status, who might also benefit from 5′-Aza treatment.

## Methods

### Patients.

AML patients with an abnormal chromosome 4 (UPN01–UPN30) were recruited to the SAL AML registry biobank in Dresden (Germany) (institutional review board number EK98032010).

Patients over 65 years of age with newly diagnosed *TET2*-mutant AML (UPN31–UPN80) were enrolled in the PETHEMA-FLUGAZA phase III clinical trial (ClinicalTrials.gov NCT02319135), as previously described (13).

### BM morphological assessment of the AML index case (UPN01).

For morphological analyses at AML presentation and during follow-up, smears were prepared from BM aspirates, stained with Giemsa, and visualized according to routine diagnostic protocols.

### Cytogenetic analyses of UPN01.

G-banding analysis of metaphase chromosomes from short-term cultures established from presentation BM aspirate was performed using well-established techniques. Interphase FISH was performed using the XL *TET2* kit (Metasystems). Spectral karyotyping (SKY) was performed using the SKYPaint probe mixture kit (Applied Spectral Imaging) according to the manufacturer’s protocol, with the exception that the hybridization time was extended from 2 to 3 days.

### AML cell lines and culture.

HEL, THP-1, HL-60, AML2, Kasumi, MV4-11, AML3, U937, SKM1, and NB4 AML cell lines were obtained from DSMZ. THP-1 shRNA-mediated TET2-knockdown cells (and parental cells) were obtained from the University of California, San Francisco. KG-1 AML cells were a gift from Ross Levine at the Memorial Sloan Kettering Cancer Center, New York, New York, USA. All AML cell lines were maintained in complete medium (CM) (RPMI-1640 [Invitrogen, Thermo Fisher Scientific] with 10% FBS and 50 μg/mL penicillin/streptomycin) at 37°C in a humidified 5% CO_2_ incubator. The identity of AML cell lines was confirmed by short tandem repeat profiling (NewGene), and cell cultures were regularly tested for mycoplasma using a MycoAlert kit (Lonza).

### CRISPR/Cas9 TET2 targeting.

CRISPR/Cas9 *TET2*-targeted HEL cells were generated using a 1-vector system: pLV-U6-gRNA/EF1a-puro-2A-Cas9-2A-GFP in lentiviral particles (Sigma-Aldrich) with the sgRNA sequence 5′GTTTGGTGCGGGAGCGAGC3′ targeting *TET2* exon 6 (*TET2* transcript ID ENST00000540549.5). Lentiviral particles were incubated with HEL cells (at MOI of 2) in CM supplemented with 8 μg/mL hexadimethrine bromide and centrifuged at 800*g* for 30 minutes at 32°C. Transduced cells were selected by culture in CM supplemented with 2 μg/mL puromycin, then subsequently cloned by plating in soft agar (CM supplemented with 0.2% agarose). DNA was extracted from cell clones using a QIAamp DNA Micro Kit (QIAGEN), and *TET2* mutation was confirmed by Sanger sequencing of exon 4 as described below (Supplemental Table 9). Control cell clones (with monoallelic *TET2* mutation) were derived from parental HEL cells transduced with virus carrying an empty vector. All the clones used in experiments were generated independently, and there is some heterogeneity in expression profiles and phenotype. Moreover, it should be noted that several independent cell clones were generated for each *TET2* genotype, and not every clone was used in every experiment.

### Nucleic acid preparation.

DNA was extracted from BM mononuclear cells (BMMNCs), peripheral blood (PB), or methanol/acetic acid–fixed cells using an appropriate QIAGEN kit or from saliva using an Oragene kit (DNA Genotek).

### SNP array genotyping.

SNP array genotyping was performed on DNA from BMMNCs using the OmniExpressExome (v1.4) platform and analyzed using GenomeStudio 2.0.3 (Illumina) with genotype, minor (B) allele frequency (B/[A + B]), and log R ratio at each locus calculated using standard parameters (GenCall Threshold 0.15). SNP coordinates are based on human genome build 37. Regions of copy number loss were identified manually based on interrogation of log R ratios and B allele frequencies.

### Whole-exome sequencing.

Exome capture (using Agilent Technologies SureSelect Protocol v1.2), library preparation, and sequencing of pooled DNA samples (from PB or saliva) were carried out by Oxford Gene Technology on the Illumina HiSeq 2000 platform. Reads were mapped to human genome build 37 (hg19) using the Burrows-Wheeler Aligner–MEM package (53), and local realignment of mapped reads around potential insertion/deletion (indel) sites was carried out using Genome Analysis Toolkit (GATK; v1.6) (54). Duplicate reads were marked using Picard (v1.98) and excluded from analysis. SNPs and indels were called using GATK HaplotypeCaller, with SNP novelty determined against dbSNP release 135. Variants were annotated with gene data from Ensembl. A read depth of at least 20× was achieved for a minimum of 95.61% of on-target regions.

### RNA sequencing and differential gene expression analysis.

Total RNA was extracted using the RNeasy Micro Kit (QIAGEN) and quantified using a Qubit 2.0 Fluorometer with Qubit RNA BR assay kit (Thermo Fisher Scientific). Quality control, library preparation, and sequencing on the NextSeq 550 platform (Illumina) were performed by Edinburgh Clinical Research Facility.

Sequencing reads were mapped to human genome build 37 (hg19) and annotated using STAR aligner (55). Aligned reads were summarized over gene features using the Rsubread package (56) (using featureCounts function) in R (v3.5.1). Read counts were normalized by expressing as CPM. Gene level differential expression analysis was performed on normalized read counts using DESeq2 (version 1.16.1) (57). Resulting *P* values were adjusted to control for the FDR (5%) (59), and significantly differentially expressed genes were defined as those with FDR-adjusted *P* < 0.05 and |log_2_FC| ≥ 0.3.

### Illumina 450K arrays and differential methylation analysis.

DNA was extracted from HEL cell clones using a DNA Mini Kit (QIAGEN) and sent for processing and hybridization to Infinium HumanMethylation450K BeadChips (Illumina) by Eurofins Genomics. Data processing and analysis were performed according to an established workflow (59). Specifically, raw intensity data files containing methylated (M) and unmethylated (U) intensity measurements were imported into R, and the minfi Bioconductor package (60) was used to calculate detection *P* values (*detP*), normalize data (using the preprocessFunnorm function), and generate β (β = M/[M+U+100]) and M (M = log_2_[M/U]) values for individual CpG probes. Poorly performing probes (*detP* < 0.01) and those interrogating SNPs were removed, leaving 410,811 probes in the final data set. The limma Bioconductor package (61) was used to identify significantly differentially methylated probes based on *TET2* mutation status (monoallelic versus biallelic) using M values. Resulting *P* values were adjusted to control for FDR (5%) (58), and significantly differentially methylated CpGs were defined as those with FDR-adjusted *P* < 0.05 and |log_2_FC| ≥ 2. Unsupervised hierarchical clustering based on M values was performed in R with scaling by SD.

### TET2, NPM1, and FLT3 mutation analysis.

Whole-gene *TET2* mutation analysis of the SAL abnormal chromosome 4 cases was performed by the MLL Munich Leukemia Laboratory on DNA from fixed BMMNCs via the generation of 27 exon-specific amplicons using the FastStart High Fidelity PCR System kit (Roche Applied Science, MilliporeSigma) as previously described (62, 63). Mutation of *TET2* exon 3 in the index AML case (UPN01), as well as *NPM1* exon 11 and *FLT3* exon 14, was confirmed by Sanger sequencing. PCR reactions consisted of 0.5 units ThermoPrime Taq DNA polymerase with 1× ReddyMix PCR buffer (Thermo Fisher Scientific), 1.5 mM MgCl_2_, 10 pmol primers, 0.2 mM (each) dNTPs (Invitrogen, Thermo Fisher Scientific), and 100 ng template DNA in a total volume of 20 μL. Primer sequences and thermal cycling conditions for individual amplicons are shown in Supplemental Table 9. PCR products were purified using the QIAquick PCR Purification Kit (QIAGEN) and sequenced using the indicated primers (Supplemental Table 9) by Source BioScience. Mutation in *TET2* was determined on the PETHEMA-FLUGAZA AML clinical trial patients from whole-exome sequencing, as previously described (13).

### Western immunoblotting.

Cellular proteins were extracted using PhosphoSafe reagent (MilliporeSigma) and quantified by Pierce BCA assay (Thermo Fisher Scientific). Proteins were separated using Novex NUPAGE 3%–8% Tris-acetate gels (Invitrogen, Thermo Fisher Scientific), transferred to nitrocellulose membranes, and immunoblotted according to routine techniques. Antibodies used were TET2 (Mab-179-050; Diagenode), ABCB1 (G-1; Santa Cruz Biotechnology), LSM8 (F-8; Santa Cruz Biotechnology), α-tubulin (T9026; Sigma-Aldrich), cleaved PARP (mAB 9541; Cell Signaling Technology), and GAPDH (0411; Santa Cruz Biotechnology). HRP-conjugated secondary antibodies were from Agilent Technologies and included goat anti-mouse immunoglobulin–HRP (P044701-2) and goat anti-rabbit immunoglobulin–HRP (P044801-2). Protein quantification was performed on immunoblots using the Fuji LAS-300 image analysis system (Raytek).

### Cell proliferation, drug sensitivity, and CE assays.

Cytotoxic agents were purchased from Sigma-Aldrich. Ara-C was reconstituted in DMSO and daunorubicin or 5′-Aza in distilled H_2_O, and aliquots were prepared and stored at –80°C. Stocks were diluted in CM immediately prior to use in cytotoxicity assays.

To compare cell proliferation between parental and CRISPR/Cas9-mutated HEL clones, exponentially growing cells were seeded at low density (2 × 10^4^ cells/mL) in CM and counted using a hemocytometer at regular intervals up to 192 hours postseeding. Cell growth at each time point was calculated relative to initial seeding density. Two-way ANOVA was used to test for significant differences in relative cell growth based on *TET2* mutation status.

For drug sensitivity experiments, cells were incubated in CM supplemented with appropriate concentrations of cytotoxic agent (5′-Aza, daunorubicin, or Ara-C) or relevant VC for 96 hours, after which viable cells were identified by trypan blue dye exclusion and counted using a hemocytometer. Survival fractions were determined at each drug concentration relative to VC-treated controls. Two-way ANOVA was used to test for significant differences in drug sensitivity based on *TET2* mutation status. Inhibition of proliferation in drug-treated cultures was compared with VC-treated cultures and used to calculate the IC_50_ and IC_90_ values in GraphPad Prism (6.0.2, GraphPad Software).

For determination of CE, exponentially growing cells were seeded in soft agar (CM supplemented with 0.2% agarose) supplemented with cytotoxic agent (5′-Aza, daunorubicin, or Ara-C) or VC. Macroscopically visible colonies were counted on day 30, and CE was calculated relative to number of cells initially seeded. Student’s *t* tests (2-tailed) were used to identify significant differences in CE based on *TET2* mutation status.

To determine the effect of ABCB1 inhibition on 5′-Aza sensitivity, cells were incubated in CM supplemented with increasing doses of 5′-Aza and an ABCB1 inhibitor (verapamil or tariquidar) or VC. After 96 hours of incubation, viable cells were identified using CellTiter-Glo Luminescent Cell Viability Assay (Promega), and the surviving fraction was determined at each drug concentration relative to VC-treated controls. The resulting dose-response matrix was used to calculate drug synergy using SynergyFinder 2.0. Student’s *t* test (2-tailed) was used to identify significant differences in synergy scores based on *TET2* mutation status.

All assays were performed in triplicate at a minimum and means ± SD were calculated.

### Generation of 5′-Aza–resistant clones.

Exponentially growing HEL AML cells were seeded in soft agar (CM supplemented with 0.2% agarose) supplemented with 10 μM 5′-Aza (which corresponds to 90%–95% cytotoxicity). Colonies were picked after 28 days and were subsequently expanded and maintained in CM supplemented with 5′-Aza to establish putative 5′-Aza–resistant clones. Following expansion each cell clone was tested for sensitivity to 5′-Aza along with the parental cell line from which the 5′-Aza–resistant clone was developed. Student’s *t* test (2-tailed) was performed to test for significant differences in IC_50_ values between 5′-Aza–resistant clones and parental cells.

For determination of ABCB1 protein expression in 5′-Aza–resistant clones, Western immunoblotting was performed, and the resulting ABCB1 band intensities in 5′-Aza–resistant clones were normalized to the ABCB1 band intensity in the respective parental cells. Student’s *t* test (2-tailed) was performed to test for significant differences in ABCB1 protein expression between 5′-Aza–resistant clones and parental cells.

### Flow cytometry for cell cycle analysis.

HEL AML cells were treated with 5′-Aza and sampled at 24 and 48 hours by fixation in 70% ethanol. Fixed cells were incubated in 50 μg/mL propidium iodide and 20 μg/mL RNase in PBS for 30 minutes in the dark. Cellular DNA content was determined using a FACSCanto II flow cytometer (BD) with gating to exclude cell debris and doublets. A total of 10,000 events were acquired for each sample, and the resulting data were analyzed using FCS Express 7 software (De Novo Software).

### In vivo mouse model.

Eight-week-old male and female *Rag2^−/−^Il2rg^−/−^* (129 × BALB/c) mice from Newcastle University (64) were used for in vivo investigations in accordance with UK Home Office Project License PPL60/4552. For IF injection (on day 0), mice were anesthetized, and 5 × 10^5^ cells (HEL *TET2* monoallelic and HEL *TET2* biallelic mixed in a 1:1 ratio) in 20 μL CM were injected through the knee into the marrow cavity of the right femur. Mice were monitored for signs of disease progression and were euthanized if any tumors reached a diameter of 15 mm, or prior to this point, if any signs of animal suffering were observed (including but not limited to sustained weight loss of 15% of normal weight and reduced mobility). 5′-Aza was dissolved in sterile water and was administered via intraperitoneal (IP) injection once daily starting on day 28 for 5 days at 5 mg/kg. Control mice received sterile water only via IP administration. Mice were euthanized on day 35, and tissues (BM from right and left femurs, PB, spleen, and any detected tumors) were harvested during postmortem investigation. Genomic DNA was extracted using either a DNA Mini Kit or DNA Micro Kit (QIAGEN) as required, according to sample volume.

### TET2 allele-specific qPCR assay.

A custom TaqMan SNP genotyping assay (Applied Biosystems, Thermo Fisher Scientific) was designed using probes that differentiate between intact WT *TET2* sequence (HEL *TET2* monoallelic clones) and *TET2* sequence with a 4 bp deletion generated by CRISPR/Cas9 targeting (HEL *TET2* biallelic clones) in samples collected from mice. qPCR reactions were set up in triplicate and consisted of genomic DNA (50 ng), primers (forward: 5′GTGAAGAGAAGCTACTGTGTTTGGT3′, reverse: 5′ACAATCACTGCAGCCTCACA3′), fluorescent allele-specific probes (WT *TET2*: 5′CCAGCTCGCTCCCG3′-VIC, 4 bp deleted *TET2*: 5′-TGGCCAGCTCCCG3′-FAM), and TaqMan SNP genotyping Master Mix (Applied Biosystems, Thermo Fisher Scientific) according to manufacturer’s recommended volumes. Controls were prepared using a 1:1 mix of DNA extracted from HEL *TET2* monoallelic and HEL *TET2* biallelic cells. Thermal cycling (50°C 2 minutes, 95°C 10 minutes, followed by 40 cycles of 95°C 15 seconds, 60°C 1 minute) was performed using a 7300 Real Time PCR System (Applied Biosystems, Thermo Fisher Scientific). Detected fluorescence for the 2 probes was converted to Ct values using SDS version 1.4 software (Applied Biosystems, Thermo Fisher Scientific), and a sample was considered positive if Ct was 38 or higher for either allele. In samples where only 1 allele was amplified in all replicates (due to complete domination of 1 cell population in the sample), a Ct value of 38 was assigned to the nonamplified allele such that the sample could be included in the analysis (such samples are indicated on relevant figures). For all samples, adjusted ΔCt values (difference between WT *TET2* allele Ct and 4 bp deleted *TET2* allele Ct, adjusted by subtracting ΔCt calculated from the control DNA with 1:1 allelic ratio) were converted to inverse log_2_ values, such that a value of 1 indicated a 1:1 ratio between the 2 alleles (and hence the 2 cell populations in the sample). Inverse log_2_ [ΔCt] values were compared between 5′-Aza–treated and VC-treated mice for each tissue type using the Mann-Whitney test.

### Data availability.

Genome-wide methylation data described in Figure 2, E and F, have been deposited at the NCBI’s Gene Expression Omnibus with accession numbers GSE217940 and GSE218228. RNA-Seq gene expression data described in Figure 5, A–C, have been deposited at the Gene Expression Omnibus with accession numbers GSE218227 and GSE218228.

### Statistics.

All statistical tests were performed using GraphPad Prism, or R, in the case of large-scale array data analysis. Specific tests and corrections applied, as well as details of experimental replicates and summary statistics, are given above and in relevant figure legends. For all analyses, a *P* ≤ 0.05 was considered statistically significant unless otherwise stated.

### Study approval.

For human studies, approval was received from institutional review boards and/or ethics committees at all German and Spanish sites, and written informed consent was received from all participants prior to inclusion. In addition, written informed consent was provided for pictures appearing in the manuscript, and the record of informed consent has been retained.

For animal studies, experimental procedures were approved by the Animal Welfare Ethical Review Body at Newcastle University and the UK Home Office (London, United Kingdom) and were performed in compliance with the UK Animals (Scientific Procedures) Act 1986 and its associated Codes of Practice.

## Author contributions

FS, SEF, DN, and WYL designed experiments, generated data, analyzed data, and wrote the manuscript. HB, CE, HLB, BM, LR, DK, CD, DA, RP, ENS, CP, MF, TR, AA, MW, HA, CR, LFW, GLJ, TM, GHJ, HJM, JF, KO, MM, AR, ZB, EB, OH, TH, SV, BA, RAD, SJA, LFW, FP, PM, JML, and MB generated or collated data or reagents and/or advised on data analysis. JMA designed experiments, generated data, analyzed data, directed the research, and wrote the manuscript. FS and JMA conceived of the project and secured funding. All authors contributed to the final version of the manuscript. FS, SEF, DN, and WYL are listed as co–first authors of the manuscript, and the authorship order was determined by the drawing of lots.

## Supplementary Material

Supplemental data

Supplemental table 2

Supplemental table 3

Supplemental table 4

Supplemental table 5

Supplemental table 6

## Version 1 12/08/2022

In-Press Preview

## Version 2 01/24/2023

Electronic publication
