# Supplementary material for: Biallelic TET2 mutations confer sensitivity to 5′-azacitidine in acute myeloid leukemia
Source: JCI Insight. 2023 Jan 24;8(2):e150368. doi: 10.1172/jci.insight.150368 (PMC9977313; doi:10.1172/jci.insight.150368)
Supplement: Supplemental data [file jciinsight-8-150368-s048.pdf]

Biallelic *TET2* mutation sensitises to 5'-azacitidine in acute myeloid leukemia

Friedrich Stölzel, Sarah E. Fordham, Devi Nandana, Wei-Yu Lin, Helen Blair, Claire Elstob, Hayden L. Bell, Brigitte Mohr, Leo Ruhnke, Desiree Kunadt, Claudia Dill, Daniel Allsop, Rachel Piddock, Emmanouela-Niki Soura, Catherine Park, Mohd Fadly, Thahira Rahman, Abrar Alharbi, Manja Wobus, Heidi Altmann, Christoph Röllig, Lisa Wagenführ, Gail L. Jones, Tobias Menne, Graham H. Jackson, Helen J. Marr, Jude Fitzgibbon, Kenan Onel, Manja Meggendorfer, Amber Robinson, Zuzanna Bziuk, Emily Bowes, Olaf Heidenreich, Torsten Haferlach, Sara Villar, Beñat Ariceta, Rosa Ayala Diaz, Steven Altschuler, Lani Wu, Felipe Prosper, Pau Montesinos, Joaquin Martinez-Lopez, Martin Bornhäuser and James M. Allan

SUPPLEMENTARY MATERIALS

Figure. S1. Disease infiltration in the AML index case.

Figure. S2. Sanger sequencing of *TET2* exon 3 in the index AML case.

Figure. S3. Sanger sequencing of *NPM1* exon 11 in the index AML case.

Figure. S4. Sanger sequencing of *FLT3* exon 14 in the index AML case.

Figure. S5. Sanger sequencing of *TET2* exon 6 in HEL AML cells.

Figure. S6. Cell cycle analysis of *TET2* mutant HEL AML cells following 5'-azacitidine treatment.

Figure. S7. Validation of qPCR assay for detection of WT and CRISPR-Cas9-mutated *TET2* alleles.

Figure. S8. Preferential engraftment of TET2-null cells in the spleen of $Rag2^{-/-}$ $Il2rg^{-/-}$ mice.

Figure S9. Abrogation of TET2 expression in THP-1 and KG-1 cells affects cellular response to 5'-azacitidine.

Figure S10. Effect of ABCB1 inhibition with Verapamil on sensitivity to 5'-azacitidine in HEL AML cell clones with monoallelic or biallelic *TET2* mutation.

Figure S11. Effect of ABCB1 inhibition with Tariquidar on sensitivity to 5'-azacytidine in HEL AML cell clones with monoallelic or biallelic *TET2* mutation.

Figure. S12. High density SNP array copy number profile of chromosome 4 from leukemic blast cells of AML patients UPN25, UPN28 and UPN30.

Table S1. Karyotypes of cell lines used in the study.

Table S2. Significantly differentially methylated CpGs ($P < 0.05$) in HEL cell clones with biallelic *TET2* mutations compared to parental clones with monoallelic *TET2* mutation.

Table S3. Significantly differentially expressed genes ($P < 0.05$ and $|\text{Log}_2\text{FC}| \geq 0.3$) in HEL cell clones with biallelic *TET2* mutations compared to parental clones with monoallelic *TET2* mutation.

Table S4. Gene ontology component analysis of significantly differentially expressed genes in HEL cell clones with biallelic *TET2* mutations compared to parental clones with monoallelic *TET2* mutation.

Table S5. Significantly differential expression ($P < 0.05$) of components of the snRNP complex (GO:0097525) in HEL cell clones with biallelic *TET2* mutations compared to parental clones with monoallelic *TET2* mutation.

Table S6. Gene ontology biological pathway analysis of significantly differentially expressed genes in HEL cell clones with biallelic *TET2* mutations compared to parental clones with monoallelic *TET2* mutation.

Table S7. Clinical characteristics and demographics of AML patients with a cytogenetically discernible chromosome 4 aberration (from SAL AML Biobank).

Table S8. Clinical characteristics and demographics of AML patients with *TET2* mutation enrolled in the PETHEME FLUGAZA phase 3 clinical trial.

Table S9. Primer sequences and PCR reaction conditions for Sanger sequencing of genes of interest.

Figure. S1

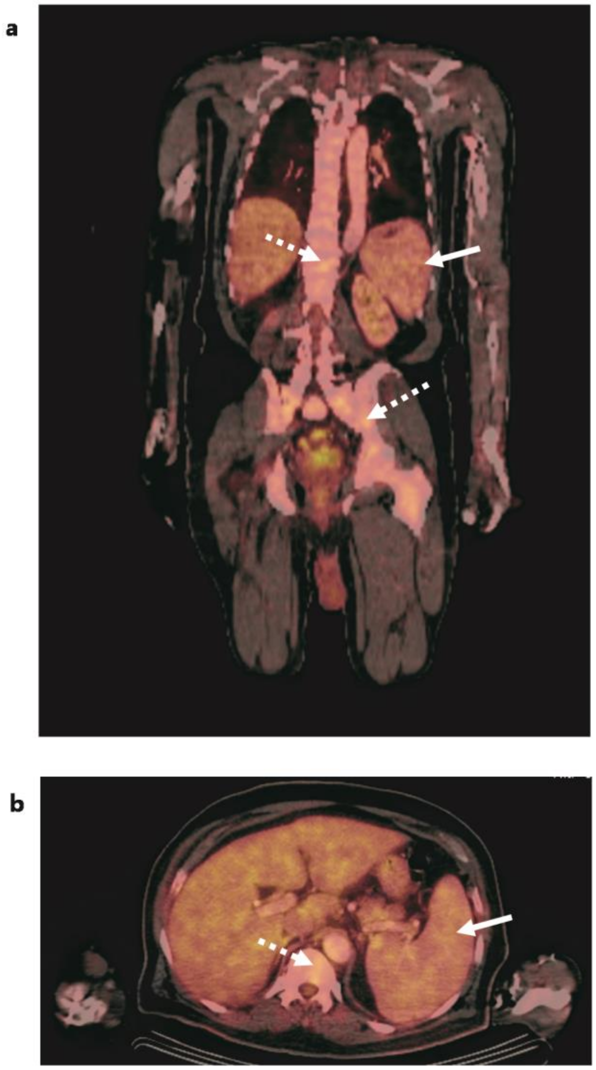

Figure. S1. Disease infiltration in the AML index case.

Coronal (**a**) and transversal (**b**) fused multiplanar reconstruction of ^{18}Fluorodesoxy-Glucose Positron Emission Tomography – Computed Tomography imaging of the index AML patient (UPN01) at diagnosis, prior to induction chemotherapy. Splenomegaly (measuring 16.5×11×5.5 cm) is indicated by solid arrows. Enhanced hypermetabolic and focal uptake in the vertebrae and pelvic bone (dotted arrows) corresponds to the AML diagnosed in bone marrow and peripheral blood of the patient. Images courtesy of Professor Jörg Kotzerke (Technical University of Dresden, Germany).

Figure. S2

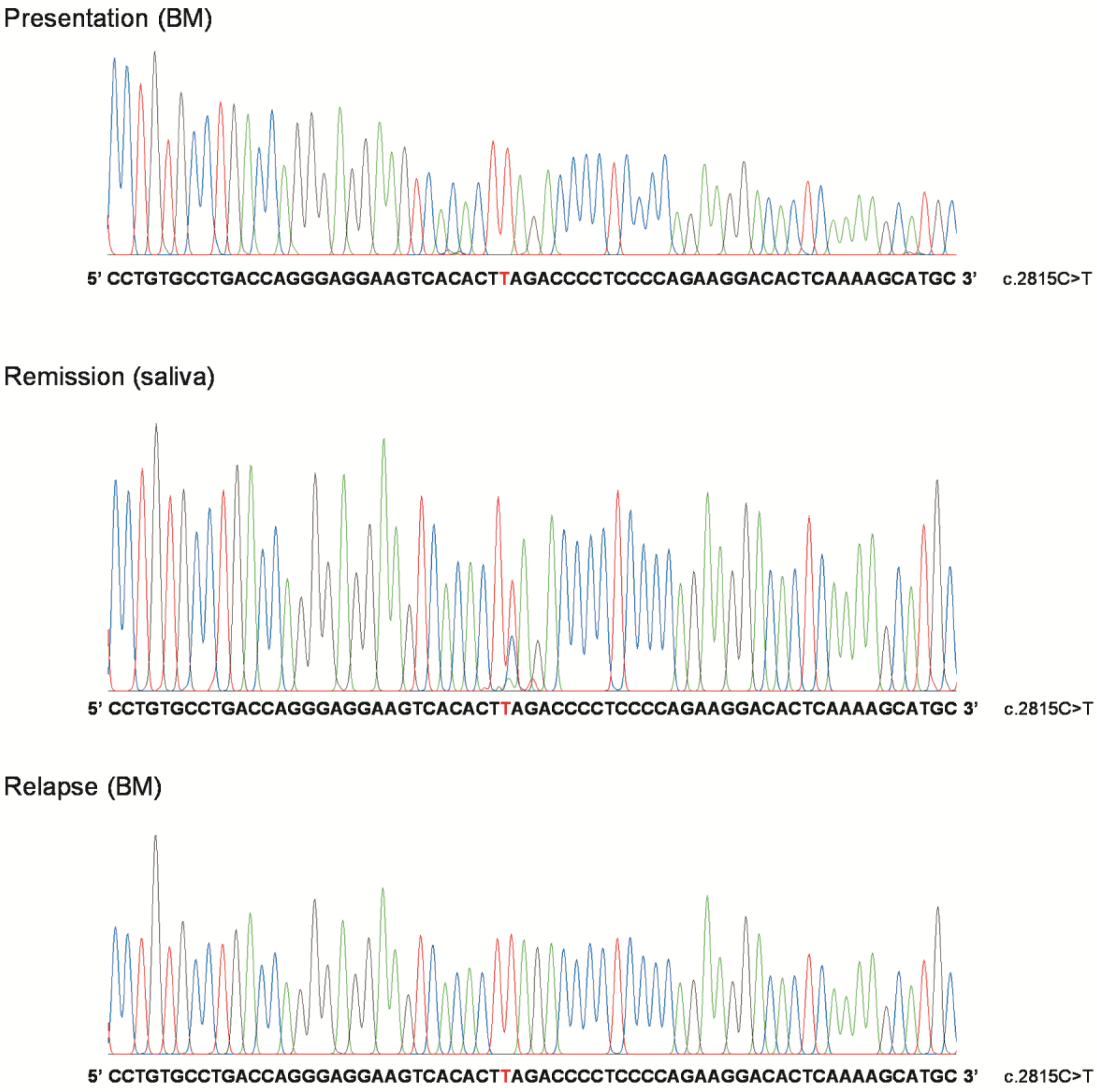

Figure. S2. Sanger sequencing of *TET2* exon 3 in the index AML case.

Sanger sequencing results for index AML patient at presentation (top), remission (middle) and relapse (bottom). A 65 bp section of forward read of *TET2* exon 3 surrounding the mutated base is shown in each case. The mutated base is shown in red. BM, bone marrow.

Figure. S3

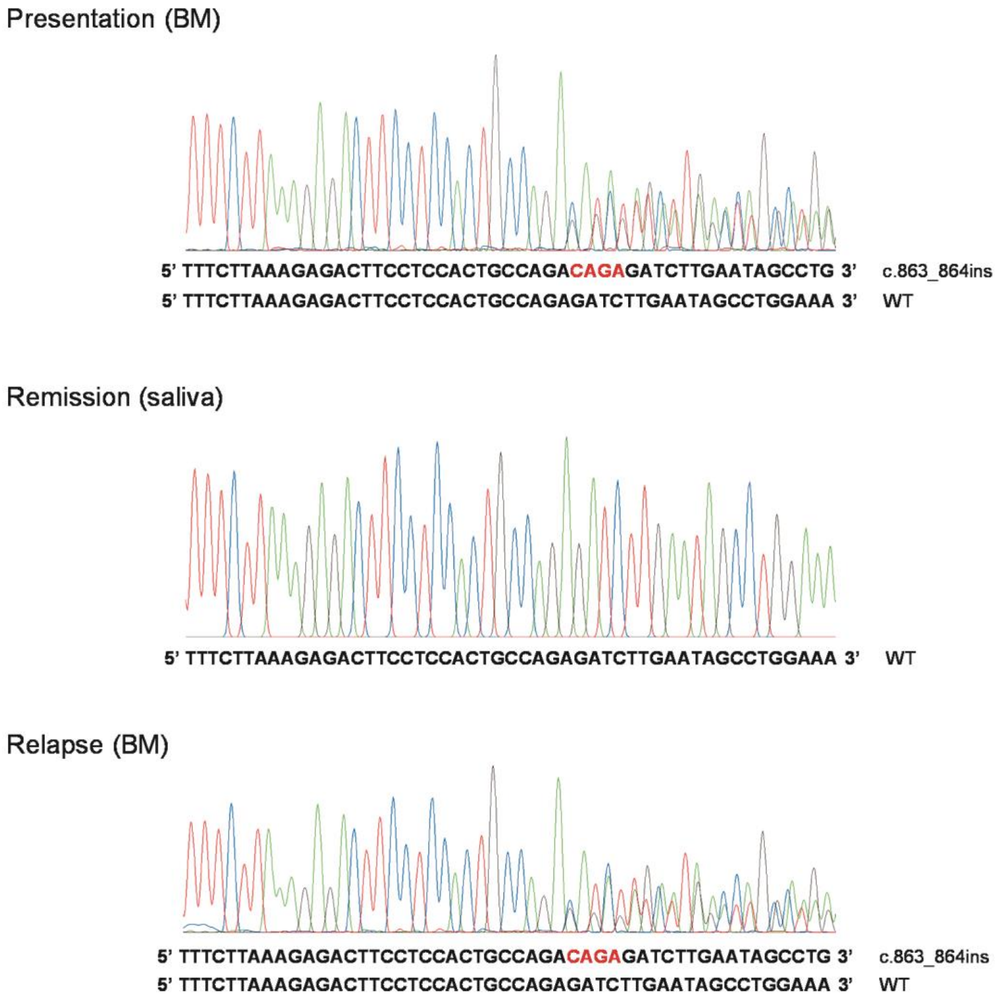

Figure. S3. Sanger sequencing of *NPM1* exon 11 in the index AML case.

Sanger sequencing results for index AML patient at presentation (top), remission (middle) and relapse (bottom). A 50 bp section of reverse complement read of *NPM1* exon 11 surrounding the insertion site is shown in each case. The 4 bp inserted sequence is shown in red. BM, bone marrow.

Figure. S4

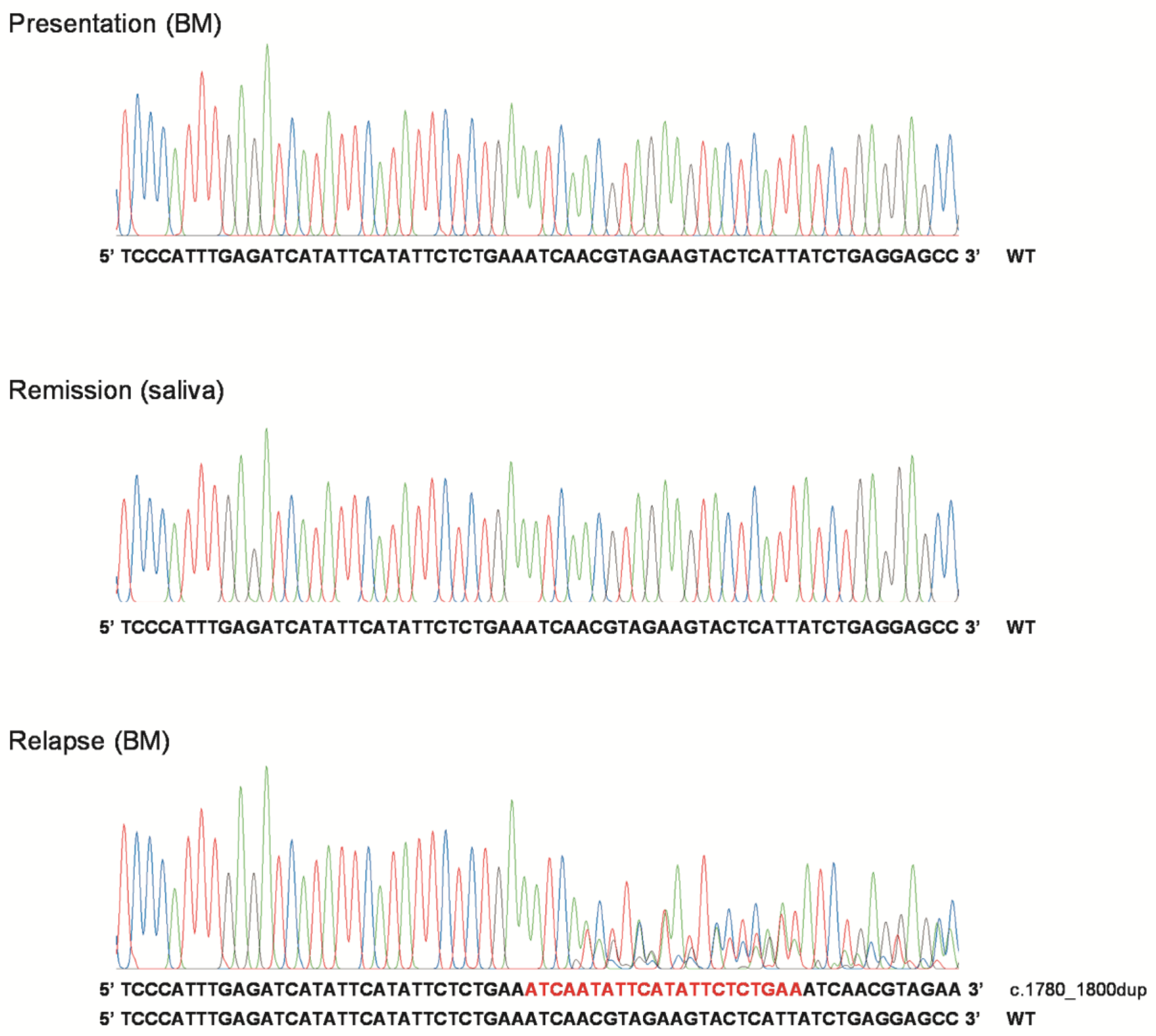

Figure. S4. Sanger sequencing of *FLT3* exon 14 in the index AML case.

Sanger sequencing results for index AML patient at presentation (top), remission (middle) and relapse (bottom). A 65 bp section of reverse complement read of *FLT3* exon 14 surrounding the duplication is shown in each case. The 21 bp duplicated sequence is shown in red. BM, bone marrow.

Figure. S5

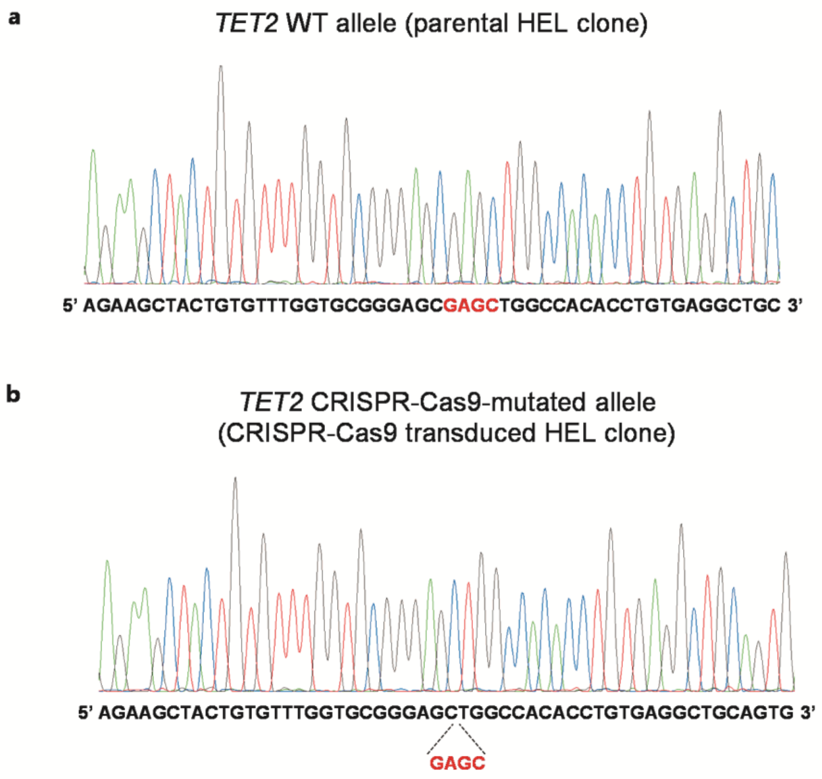

Figure. S5. Sanger sequencing of *TET2* exon 6 in HEL AML cells.

(**a**) Sanger sequencing of *TET2* exon 6 in a representative parental *TET2* HEL cell clone with WT sequence. (**b**) Sanger sequencing of *TET2* exon 6 in a *TET2* CRISPR-Cas9 transduced HEL cell clone with a 4bp deletion (highlighted in red).

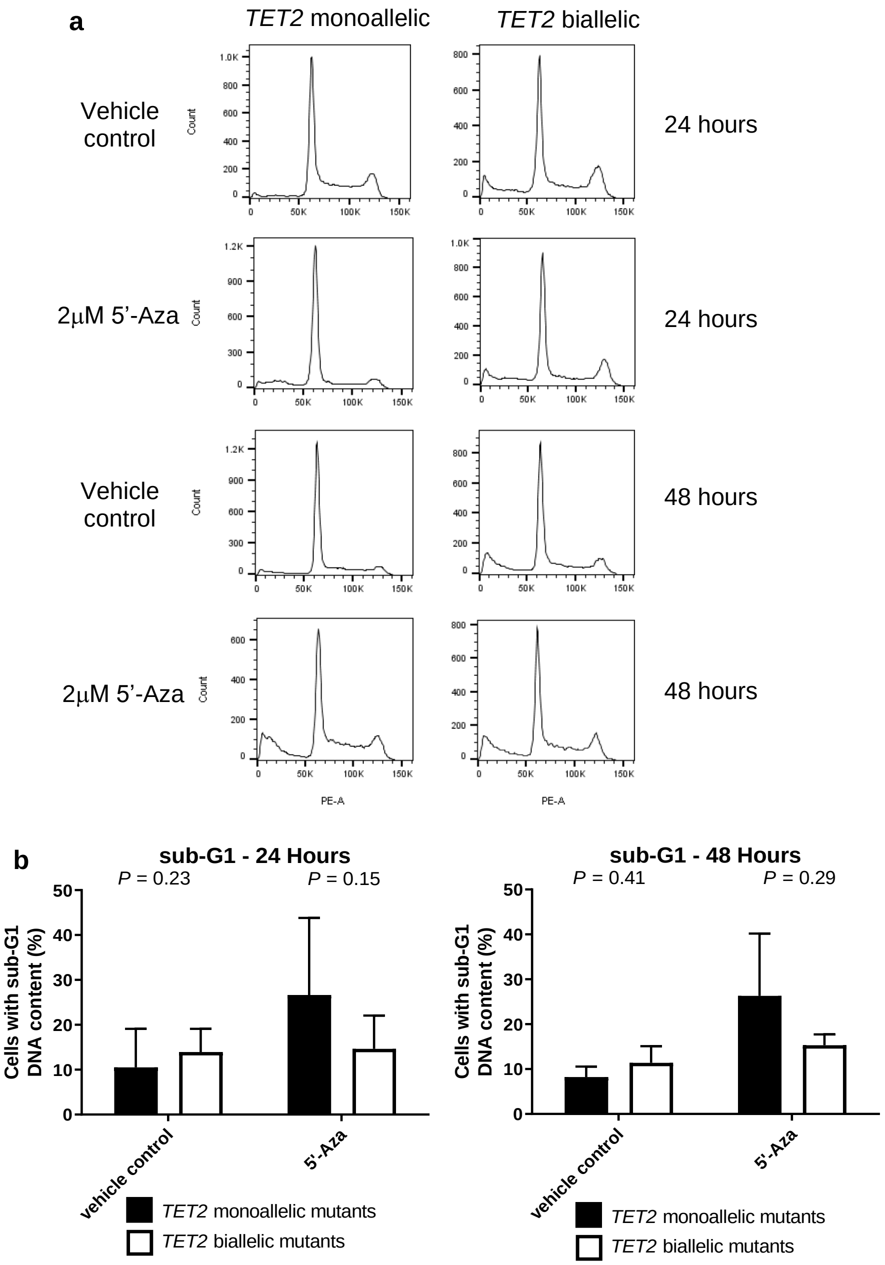

Figure. S6. Cell cycle apoptosis analysis of *TET2* mutant HEL AML cells following 5'-azacitidine treatment.

(**a**) HEL AML cell clones with monoallelic (left) or biallelic (right) *TET2* mutation were cultured with 2 μM 5'-azacitidine or vehicle control. Cellular DNA content (PE-A) at 24 and 48 hours incubation was determined using flow cytometry of propidium iodide-stained cells to identify cells with sub-G1 DNA content as a marker of apoptosis. **(b)** *TET2* monoallelic mutant (closed symbols) and *TET2* biallelic mutant (open symbols) HEL AML cells with sub-G1 DNA content were quantified at 24 hours (left panel) and 48 hours (right panel) following incubation with 2 μM 5'-azacitidine or vehicle control. Data are derived from 3 independent experiments. 5'-Aza, 5'-azacitidine; Count, cell count; PE-A, phycoerythin-area.

Figure. S7

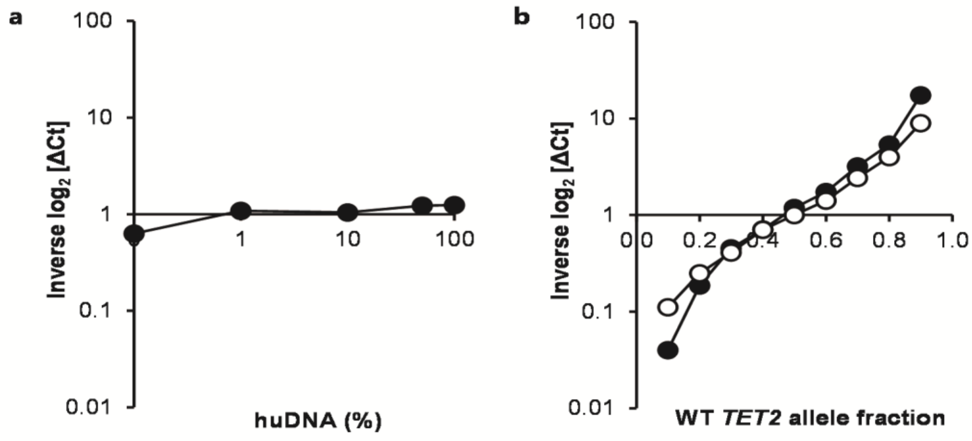

Figure. S7. Validation of qPCR assay for detection of WT and CRISPR-Cas9-mutated *TET2* alleles.

(**a**) Using a custom designed Taqman SNP Genotyping assay (see Methods), human DNA (huDNA) (from an equal mix of HEL *TET2* monoallelic and HEL *TET2* biallelic cells) was amplified in the presence of murine DNA in varying proportions and relative expression of the WT versus the mutated *TET2* allele (expressed as inverse Log_2 [ΔCt]) was calculated. Equal amplification of both alleles is represented by a value of 1, whereas dominance of the WT or mutant allele is represented by inverse Log_2 [ΔCt] values >1 or <1, respectively. As shown, amplification of huDNA was robust even when present at only 0.1% in murine DNA. Furthermore, no significant amplification bias of either WT or CRISPR-Cas9-mutated *TET2* alleles was observed. Amplification of either allele did not occur when only murine DNA was present in the reaction, confirming the assay to be specific for huDNA. Data represents the mean of triplicate reactions. (**b**) HEL *TET2* monoallelic and HEL *TET2* biallelic cells were mixed in varying ratios and extracted DNA was amplified using the custom Taqman SNP Genotyping assay as above. Closed circles demonstrate the actual experimentally-derived data (shown are means of triplicate reactions) and open circles represent the hypothetical data, assuming perfect amplification of both alleles at the designated ratios. As shown, there was no significant allele amplification bias when clones were mixed at ratios between 1:4 (WT allele fraction = 0.2) and 4:1 (WT allele fraction = 0.8). However, at ratios of 1:9 (WT allele fraction = 0.1) and 9:1 (WT allele fraction = 0.9) there was modest amplification bias in favour of the more abundant allele, presumably due to allelic drop-out of the less abundant allele.

Figure. S8

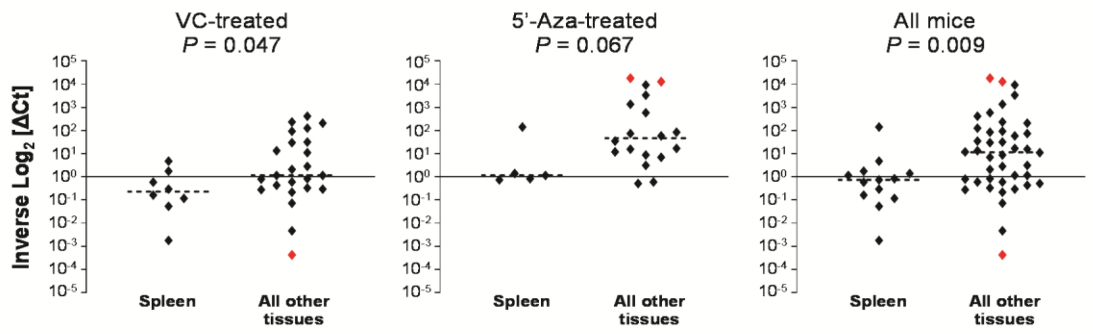

Figure. S8. Preferential engraftment of *TET2*-null cells in the spleen of *Rag2*$^{-/-}$*Il2rg*$^{-/-}$ mice.

HEL *TET2* monoallelic and HEL *TET2* biallelic cell clones were co-injected in a 1:1 ratio into the femurs of *Rag2*$^{-/-}$*Il2rg*$^{-/-}$ mice. Treatment with 5-Aza (5mg/kg daily for 5 days) or VC was initiated on day 28 (post-injection) and tissues were harvested on day 35 for analysis by custom *TET2* allele-specific qPCR assay. Shown are inverse Log_2 [ΔCt] values which represent relative expression of the WT versus the 4bp deleted *TET2* allele in individual samples and are the means of triplicate reactions. Inverse Log_2 [ΔCt] of 1 indicates a 1:1 ratio between the WT and 4bp deleted *TET2* alleles (and hence HEL *TET2* monoallelic and HEL *TET2* biallelic clones), whereas inverse Log_2 [ΔCt] > 1 or inverse Log_2 [ΔCt] < 1 indicates dominance of the WT (HEL *TET2* monoallelic) or 4bp deleted (HEL *TET2* biallelic) allele, respectively. Red points indicate samples which were dominated entirely by one cell clone. Horizontal dashed lines represent median inverse Log_2 [ΔCt] values. *P* values comparing inverse Log_2 [ΔCt] values from spleens versus all other tissues (BM, PB and tumours) in VC-treated (left), 5'-Aza-treated (center) or all mice regardless of treatment (right) were calculated using the Mann-Whitney test. VC, vehicle control.

Figure. S9

a

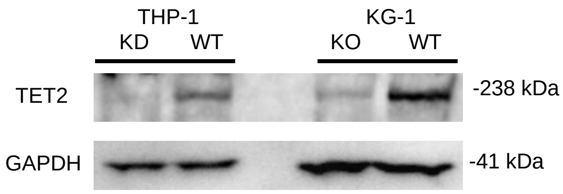

b

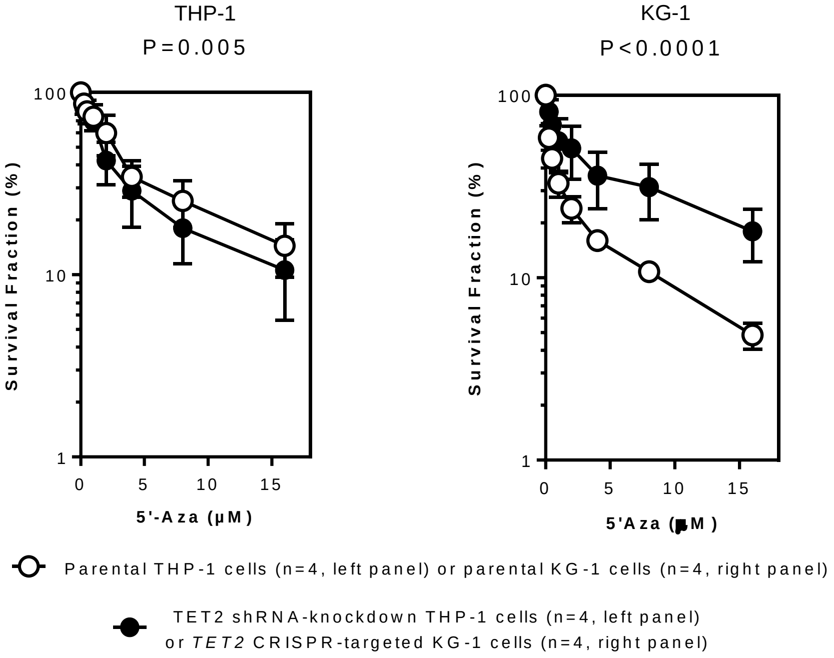

Figure. S9. Abrogation of TET2 expression in THP-1 and KG-1 cells affects cellular response to 5'-azacitidine

(a) Immunoblot showing TET2 protein expression in THP-1 cells with shRNA-mediated knockdown (KD) of TET2 and in KG-1 cells with CRISPR-targeted knockout (KD) of *TET2*. **(b)** Parental THP-1 cells (left panel, open circles), parental KG-1 cells (right panel, open circles), TET2 shRNA knockdown THP-1 cells (left panel, filled circles) and CRISPR-Cas9-targeted TET2 KG-1 cells (right panel; filled circles) were treated with 5'-Aza. Cell density (relative to respective vehicle control-treated cells) was determined after 96 hrs. Data represents mean and SD of four independent experiments. *P* values calculated by two-way ANOVA.

Figure. S10

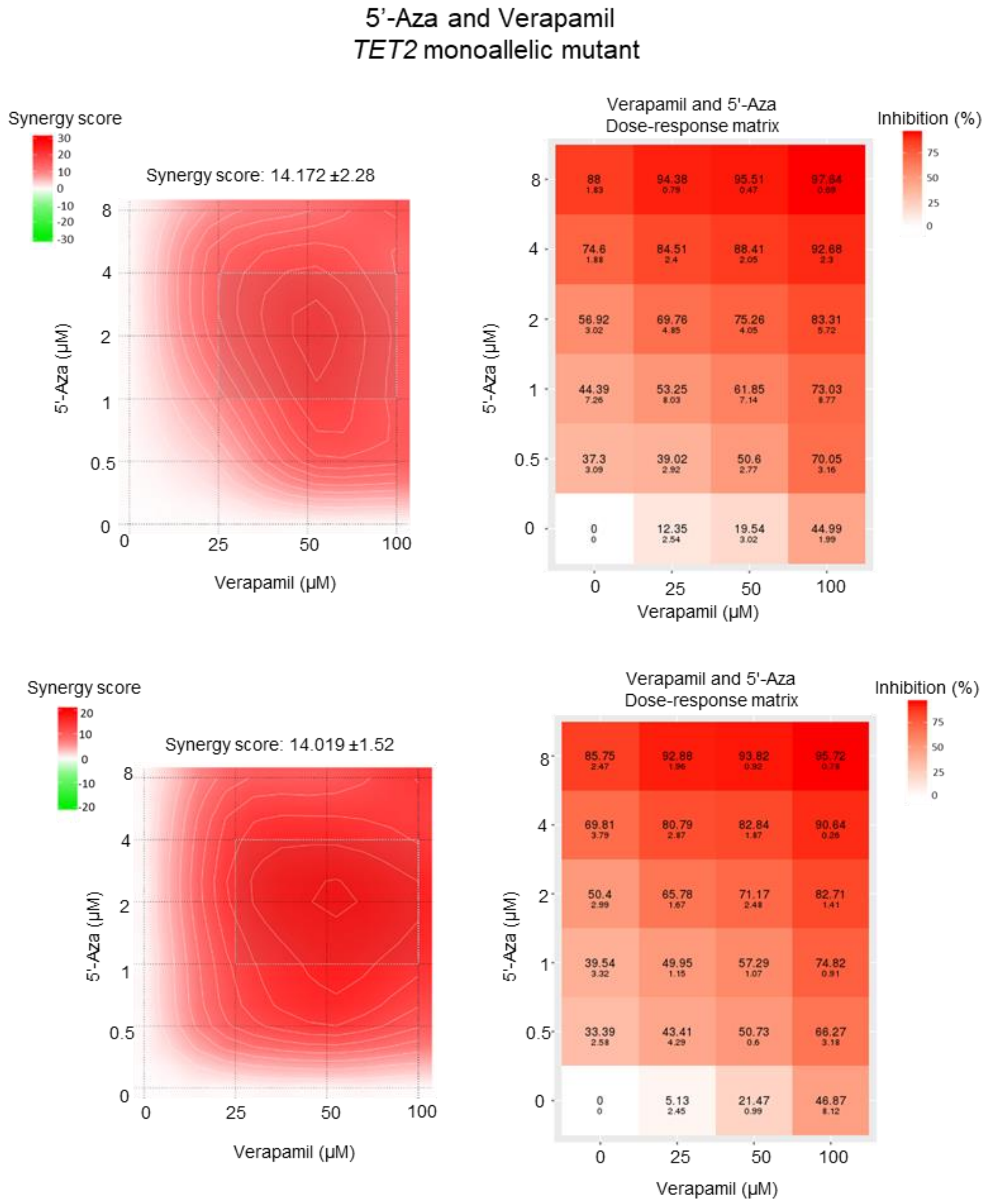

Figure S10. Effect of ABCB1 inhibition with Verapamil on sensitivity to 5’-azacitidine in HEL AML cell clones with monoallelic or biallelic *TET2* mutation (continued on next page). Synergy maps (left) show the synergy scores and the dose-response matrices (right) show HEL cell growth inhibition for 5’-Azacitidine in combination with Verapamil stratified by *TET2* mutant allele dosage (*TET2* monallelic mutant clones (N=2, this page) and *TET2* biallelic mutant allele clones (N=2, next page)).

Figure S10 (continued)

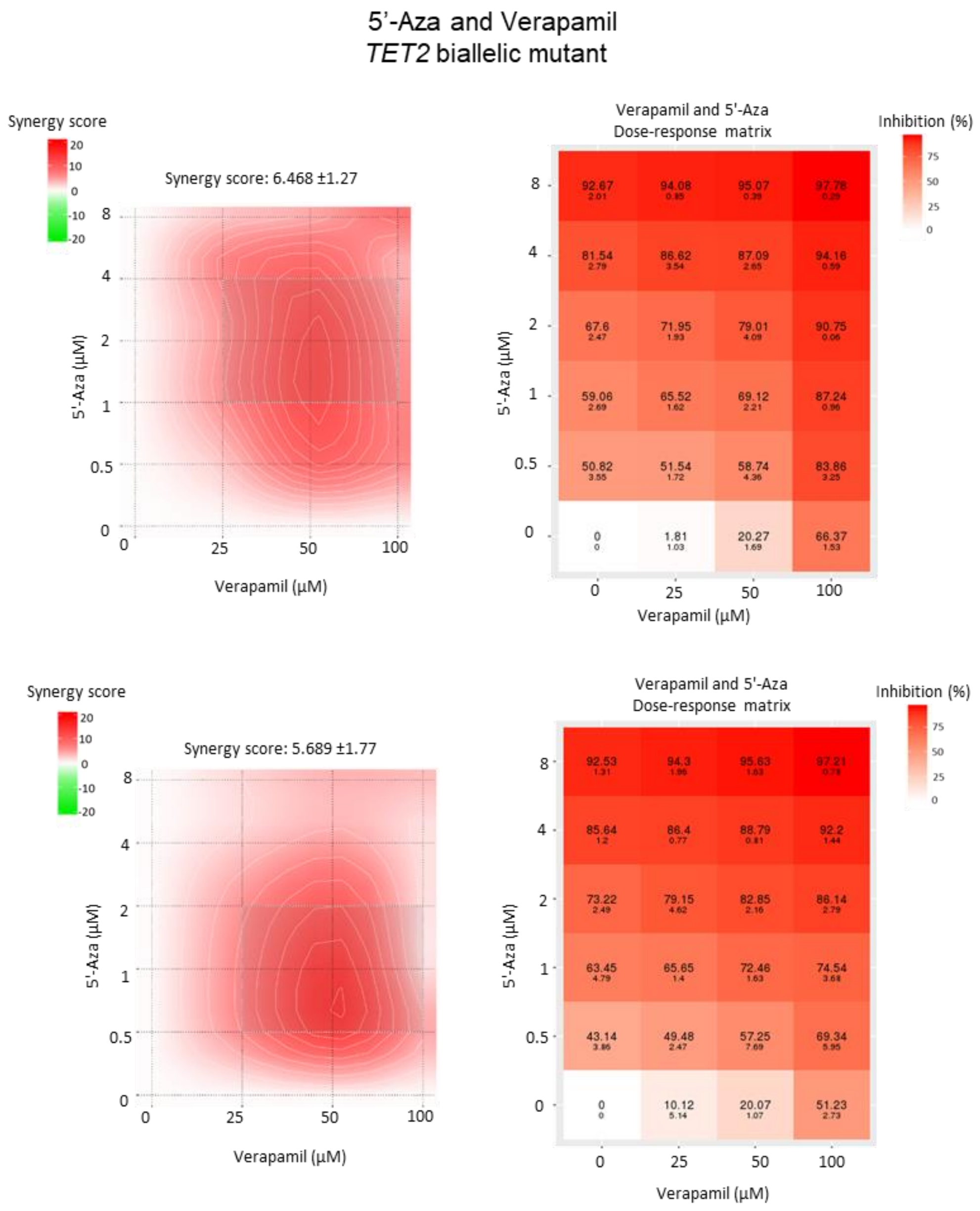

Figure S10. Effect of ABCB1 inhibition with Verapamil on sensitivity to 5'-azacitidine in HEL AML cell clones with monoallelic or biallelic *TET2* mutation (continued from previous page).

Synergy maps (left) show the synergy scores and the dose-response matrices (right) show HEL cell growth inhibition for 5'-Azacitidine in combination with Verapamil stratified by *TET2* mutant allele dosage (*TET2* monallelic mutant clones (N=2, previous page) and *TET2* biallelic mutant allele clones (N=2, this page)).

Figure S11.

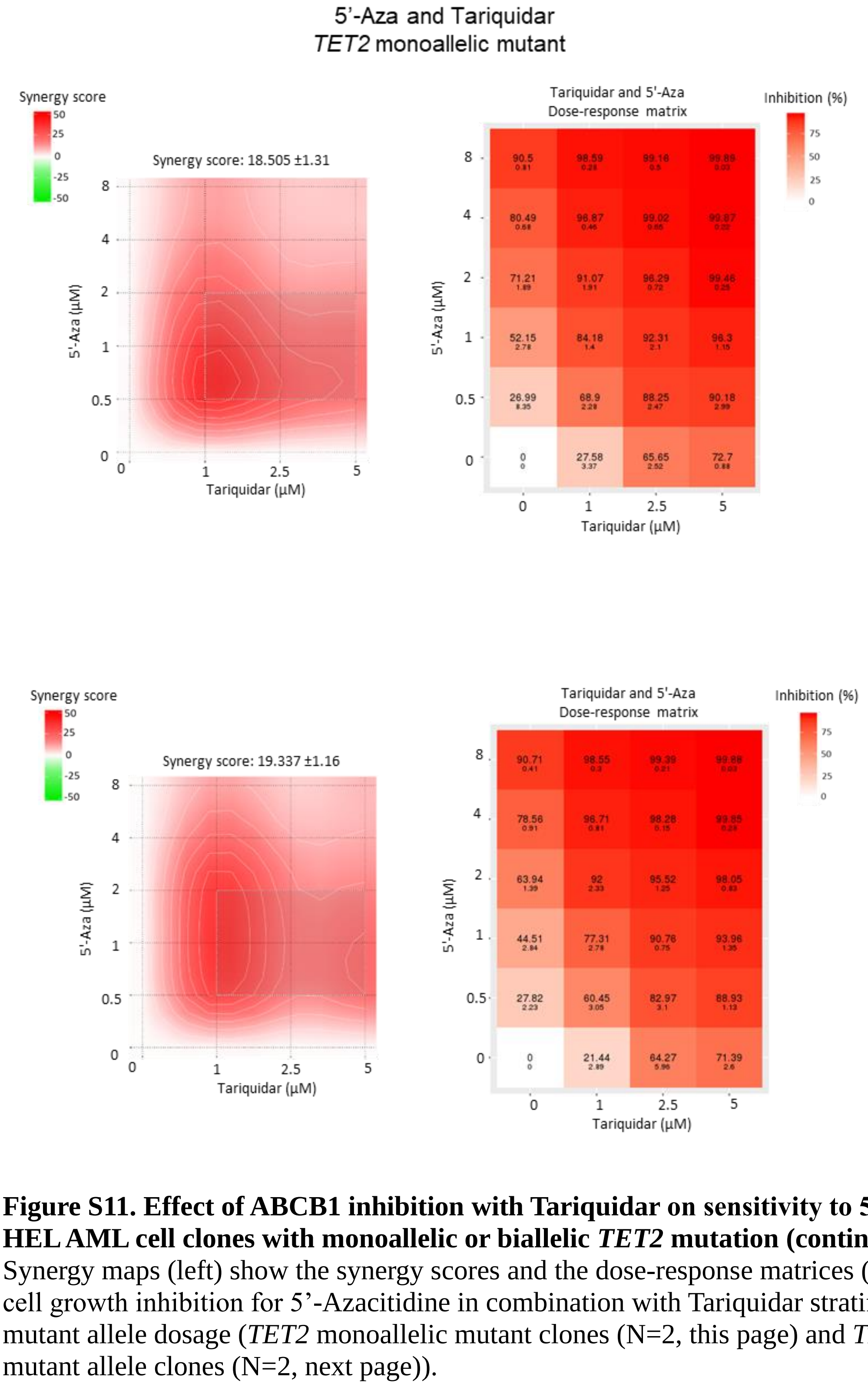

Figure S11. Effect of ABCB1 inhibition with Tariquidar on sensitivity to 5'-azacitidine in HEL AML cell clones with monoallelic or biallelic *TET2* mutation (continued on next page). Synergy maps (left) show the synergy scores and the dose-response matrices (right) show HEL cell growth inhibition for 5'-Azacitidine in combination with Tariquidar stratified by *TET2* mutant allele dosage (*TET2* monoallelic mutant clones (N=2, this page) and *TET2* biallelic mutant allele clones (N=2, next page)).

Figure S11 (continued)

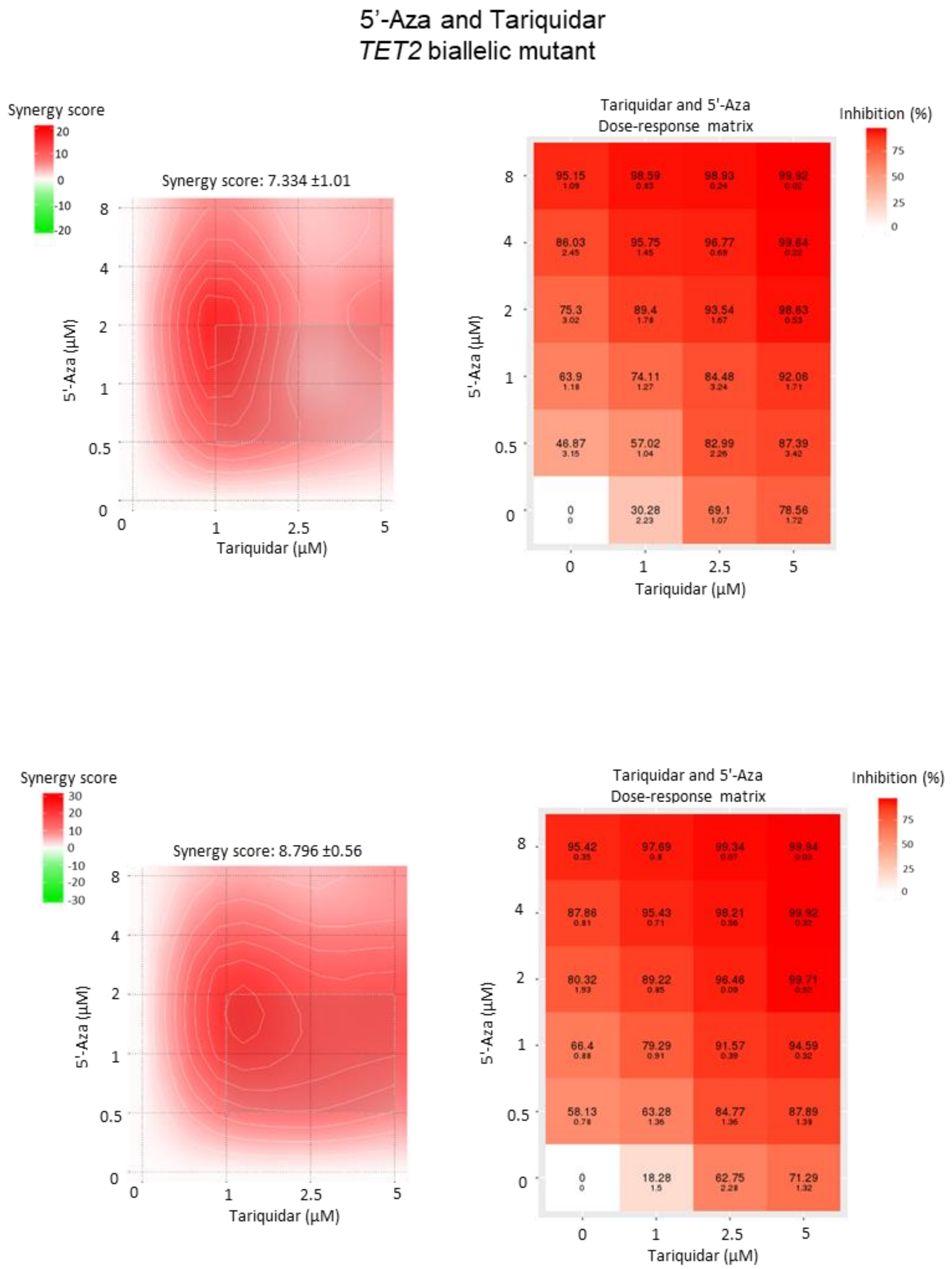

Figure S11. Effect of ABCB1 inhibition with Tariquidar on sensitivity to 5’-azacitidine in HEL AML cell clones with monoallelic or biallelic *TET2* mutation (continued from previous page).

Synergy maps (left) show the synergy scores and the dose-response matrices (right) show HEL cell growth inhibition for 5’-Azacitidine in combination with Tariquidar stratified by *TET2* mutant allele dosage (*TET2* monoallelic mutant clones (N=2, previous page) and *TET2* biallelic mutant allele clones (N=2, this page)).

Figure. S12

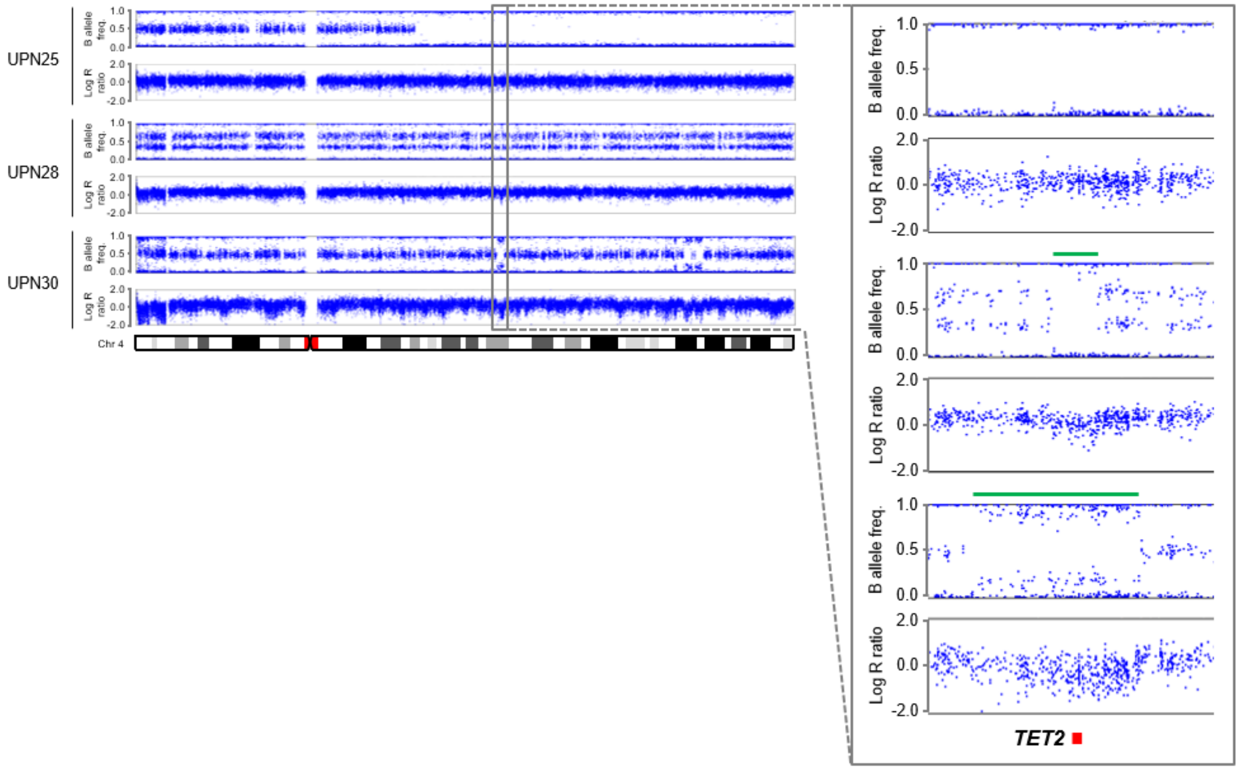

Figure. S12. High density SNP array copy number profile of chromosome 4 from leukemic blast cells of AML patients UPN25, UPN28 and UPN30.

Points represent individual SNPs which are aligned relative to their position on chromosome 4 (indicated by the ideogram below the plots). Copy number is measured as Log R ratio, with a value of 0 indicating a diploid SNP and positive and negative values indicating copy number gain and loss, respectively. B allele frequency represents the ratio of the two alleles of each SNP such that a value of 0.5 indicates allele heterozygosity and values of 0 and 1 indicate homozygosity. Inset shows expanded view of the region indicated by the grey box. Green bars highlight focal deletions within 4q24 in two of the patients. The location of *TET2* is shown below the plots.

Table S1. Karyotypes of cell lines used in the study.

Cell line	Karyotype
AML-2	48(43-49)<2n>XY, +6, +8, der(1)inv(1)(p36q31)t(1;6) (q13;p12), der(2)t(2;17)(p23;q24.1)del(2)(q14.2q36), der(3)t(1;3)(p36;p25), ins(3;2)(q21;q14.2q36), t(5;8)(q11.2;q24), der(6)t(1;6)(q31;p12)t(3;6) (q26;q24), inv(12)(p13.3q13.2), t(13;14)(q32/33;q24.2), der(17)t(2;17)(p23;q24.1)[a]
AML-3	48(45-50)<2n>X/XY, +1, +5, +8, der(1)t(1;18)(p11;q11), i(5p), del(13)(q13q21), dup(17)(q21q25)[a]
THP-1	94(88-96)<4n>XY/XXY, -Y, +1, +3, +6, +6, -8, -13, -19, -22, -22, +2mar, add(1)(p11), del(1)(q42.2), i(2q), del(6)(p21)x2-4, i(7p), der(9)t(9;11)(p22;q23)i(9)(p10)x2, der(11)t(9;11)(p22;q23)x2, add(12)(q24)x1-2, der(13)t(8;13)(p11;p12), add(?18)(q21)[a]
Kasumi-1	45<2n>X, -Y, -9, -13, -16, +3mar, t(8;21)(q22;q22), der(9)t(9;?)(p22;?), der(15)t(?9;15)((?q11;?p11)[a]
HEL	63(60-64)<3n>XYY, -2, -9, -10, -10, -11, -14, -16, -16, -17, -19, +20, +21, +2mar, del(2)(q32), t(3;6)(p13;q16), der(5)t(5;17)(q10;q10), der(6)t(1;6)(p13;p21), der(7)add(7)(p14;q32), add(8)(p21), der(9)t(9;?)(?;11)(p24;?)(?;q13), del(11)(q13), add(15)(p11), del(20)(q13), r(20)(p11q11), dup(21)(q11q22.3-qter), psu dic(22;9)t(9;?)(?;22)(p24;?)(?;p11-13)[a]
HL-60	82-88<4n>XX, -X, -X, -8, -8, -16, -17, -17, +18, +22, +2mar, ins(1;8)(p?31;q24hsr)x2, der(5)t(5;17)(q11;q11)x2, add(6)(q27)x2, der(9)del(9)(p13)t(9;14)(q?22;q?22)x2, der(14)t(9;14)(q?22;q?22)x2, der(16)t(16;17)(q22;q22)x1-2, add(18)(q21)[a]
NB4	78(71-81)<3n>XX, -X, +2, +6, +7, +7, +11, +12, +13, +14, +17, -19, +20, +4mar, der(8)t(8;?)(q24;?), der(11)t(11;?)(?->::11p15->11q22.1::11q13->22.1:), der(12)t(12;?)(p11;?), 14p+, t(15;17)(q22;q11-12.1), der(19)t(10;19)(q21.1;p13.3)x2[a]
U937	63(58-69)<3n>XXY, -2, -4, -6, +7, -9, -20, -21, +3mar, t(1;12)(q21;p13), der(5)t(1;5)(p22;q35), add(9)(p22), t(10;11)(p14;q23), i(11q), i(12p), add(16)(q22), add(19)(q13)[a]
MV4-11	48, XY, t(4;11)(q21;q23), +8, +19[b]
SKM1	43(38-43)<2n>XY, +1, -12, -14, -20, -21, t(1;19)(q21;q13), del(2)(p11), del(9)(q12), add(17)(p1?)[a]
KG1	45(42-47)<2n>X/XY, -4, +8, +8, -12, -17, -20, +2mar, der(5;17)(q10;q10)del(5)(q?11q?13), dup(7)(q12q33), del(7)(q22q35), i(8q)x2, der(8)t(6;8)(p11;q22), der(8)t(8;12)(p11;q13), der(11)t(1;11)(q13-21;p11-p13), der(16)t(?12;16)(?p13;q13/21)[a]

[a] Data from https://www.dsmz.de/collection/catalogue/human-and-animal-cell-lines/catalogue
[b] Data from https://www.lgcstandards-atcc.org/products/all/CRL-9591.aspx?geo_country=gb#characteristics

Table S2. Significantly differentially methylated CpGs (*P* < 0.05) in HEL cell clones with biallelic *TET2* mutations compared to parental clones with monoallelic *TET2* mutations.

(Excel file)

Table S3. Significantly differentially expressed genes (P_{adj} < 0.05 and $|Log_2FC| \geq 0.3$) in HEL cell clones with biallelic *TET2* mutations compared to parental clones with monoallelic *TET2* mutation identified.

(Excel file)

Table S4. Gene ontology component analysis of significantly differentially expressed genes in HEL cell clones with biallelic *TET2* mutations compared to parental clones with monoallelic *TET2* mutation.

(Excel file)

Table S5. Significantly differential expression (P_{adj} < 0.05) of components of the snRNP complex (GO:0097525) in HEL cell clones with biallelic *TET2* mutations compared to parental clones with monoallelic *TET2* mutation.

(Excel file)

Table S6. Gene ontology biological pathway analysis of significantly differentially expressed genes in HEL cell clones with biallelic *TET2* mutations compared to parental clones with monoallelic *TET2* mutation.

(Excel file)

Table S7. Clinical characteristics and demographics of AML patients with a cytogenetically discernible chromosome 4 aberration (from SAL AML Biobank).

Patient ID	Gender	Karyotype	Copy number alteration affecting 4q24 (from cytogenetics)	Age at diagnosis	ELN risk[a]	WBC count	Treatment[b]	CR	ALL-SCT[c]	Relapse	OS (months)	Status (at last follow-up)	*TET2* copy number alteration (from SNP array)	*TET2* base substitution (from Sanger sequencing)	*TET2* loss of function mutation status
UPN01 (index case)	M	46,XY,t(4;12)(q2?;q13)[12]/46,XY[10]	not discernible	76	int	38.2	DA1, AZA	no	-	yes	27.9	deceased	focal monoallelic deletion	c.2815C>T, p.Gln939*	biallelic
UPN02	M	42~44,X,-Y,der(4)t(4;12)(q21;q24),del(5)(q13q33),-6,-11,der(12)t(6;12)(q21p12),t(12;6)(q13;q21),t(6;4)(q24;q25),-13,der(15)t(11;15)(q21;p11),t(11;15)(q21;q23),der(16)t(13;16)(q14;q12),-18,der(19)t(19;20)(q12;q12),der(20)t(15;20)(q23;q11)[cp19]/46,XY[1]	negative	50	adv	6.9	DA1	yes	salvage	yes	22.8	deceased	negative	negative	
UPN03	F	46,XX,der(4)del(4)(q?11)t(4;?11)(q?11;q23q23),der(7)del(7)(q?11)t(7;?10)(q?11;p12),+8,der(10;11)(10qter-->10p12::11q23-->11q23:?11p11-->11q13::4q?21-->4qter)[8]	negative	22	adv	14.3	DA1	yes	salvage	yes	26.5	deceased	negative	negative	
UPN04	M	46,XY,der(3)inv(3)(q21q26)add(3)(p25),t(4;6)(q35;q23),del(7)(q21q31),t(8;21)(q22;q22),add(14)(q32.1),add(15)(q24)[11]/45,X,-Y,der(3)inv(3)(q21q26)add(3)(p25),t(4;6)(q35;q23),del(7)(q21q31),t(8;21)(q22;q22),add(14)(q32.1),add(15)(q24)[9]	negative	40	fav	21.9	DA1, DA2	yes	post-CR	yes	76.8	alive	negative	negative	
UPN05	F	46,XX,t(4;22)(q25;q13)[5]/46,XX[20]	negative	57	int	25.8	DA1, DA2, MAC, MAMAC	yes	salvage	yes	12.6	deceased	negative	negative	
UPN06	M	42,XY,+der(1)t(1;16),del(2),?der(3)t(3;15),?+del(4),-5,der(7)t(7;22),der(7)t(2;7;16),?i(8q),-9,der(12)t(5;12),-15,-16,-17,t(17;18),-18,-22,?ins(22;3),+mar[3]/42,XY,+der(1)t(1;16),del(2),der(3)t(3;15),?+del(4),-5,-6,der(7)t(2;7;16),der(12)t(5;12),-13,-13,	not discernible	53	adv	4.1	DA1	yes	salvage	yes	6.4	deceased	negative	negative	
UPN07	M	38-45,XY,del(1)(?q21),ins(1;4)(q?21;?),der(3)(3qter->3p21::?17q11.2->?17q21::?6->?6),t(5;17)(p10;q10),del(6)t(6;12)(p21;?),der(6)t(6;17)(p21;?),del(7)t(7;17)(p13;?)t(?3;7)(p21;q?22),+8,i(8)(q10)+1-2,-16,-17[cp14]/46,XY[1]	not discernible	51	adv	43.24	DA1	no	-	no	2.4	deceased	negative	negative	
UPN08	M	46,XY,der(5)del(5)(q31)t(4;5)(?;3)[2]/46,XY,der(5)del(5)(q31)t(4;5)(?;q31)[6]	not discernible	20	adv	34.2	DA1, DA2	no	-	no	1.6	deceased	negative	negative	
UPN09	M	46-49,XY,del(4)(q13),add(7)(q11),der(17;18)(q10;q10),der(21)t(14;21)(q12;q11),+mar1-3[cp16]/46-49,XY,+19,der(21)t(14;21)q22;q11),+22[cp4]	negative	55	adv	3.7	DA1	yes	-	no	1.2	deceased	large monoallelic deletion (*TET2* copy number < 2)	negative	monoallelic
UPN10	F	46,XX,add(4)(q31),del(4)(q24q28),-5,del(7)(q21q35),add(12)(p11),-17,-20,+1-2mar[13]/45,idem,-3,del(4)(q24q28),del(8)(q12q13),-add(12)(p11,add(18)(q12) [2]	deletion 4q24	48	adv	4.6	DA1	yes	post-CR	no	100.6	alive	large monoallelic deletion (*TET2* copy number < 2)	negative	monoallelic
UPN11	M	47,XY,del(3)(p14),der(4)t(4;5)(q26;?),der(5)t(5;12)(q14;?),der(6;21)(q10;q10),der(6)ins(6;21)(q11;q11q22),t(6;21)(p25;q11),der(7)t(7;12)(q11;q?),der(12)t(7;12)(?;q21),t(4;7)(?;?),r(21)ins(21;18),+r(21)ins(21;18)[cp13]/46,XY[3]	not discernible	53	adv	2.41	DA1	yes	salvage	yes	4.7	deceased	negative	negative	
UPN12	M	47,XY,+21[12]/47,XY,+21,del(4)(q?)[3]/47,XY,del(13)(q?)[5]/47,XY,+21,del(4)(q?),del(13)(q?)[1]	not discernible	45	fav	277	DA1, DA2	yes	salvage	yes	131.8	alive	negative	negative	

UPN13	F	47~49,XX,del(1)(p13p22),-4,del(5)(q13q33),+8,+8,add(11)(q2?3),der(12)t(4;12)(q21;p1?3),-13,-14,?der(16),-17,-18,-19,+3-7mar[cp10]	monosomy 4	72	adv	9	DA1	no	-	no	0.3	deceased	negative	negative	
UPN14	M	46,XY,t(4;14)(q11;q32)[12]/46,XY[1]	negative	64	int	5.9	DA1, DA2, MAMAC	yes	-	yes	66.7	deceased	negative	negative	
UPN15	M	46,XY,t(4;21)(q11;q11)[23]	negative	34	int	21.7	MAV, MAMAC	yes	post-CR	no	94.4	alive	negative	negative	
UPN16	F	45,XX,?t(4;10)(p11;p15),del(5)(q13q33),-7,del(12)(p13)[15]	negative	65	adv	43	DA1	no	-	no	4.6	deceased	negative	negative	
UPN17	M	45~52,XY,der(1)t(1;3)(p22;p11),der(3)t(1;3;5)(p22;p11;q11),del(6)(q21),?del(8)(q22),-12,del(13)(q13q22),der(20)t(4;20)(q13;q11),+mar,inc[cp30]	negative	73	adv	2.1	DA1	no	-	no	2.4	deceased	negative	negative	
UPN18	M	43~44,XY,del(1)(q32),der(3)(p?),del(4)(q?),del(5)(q13q31),-7,add(8)(p23),i(11)(q10),add(17)(q25),add(22)(q13),add(22)(q13),inc[cp27]	not discernible	74	adv	28.9	DA1	no	-	no	0.6	deceased	large monoallelic deletion (*TET2* copy number < 2)	negative	monoallelic
UPN19	F	46,XX,der(4)(q31),del(9)(q13q22),-12,del(13)(q14q22),der(14)(q13),del(20)(q11),+mar[cp19]/46,XX [2]	negative	62	adv	1.6	DA1	yes	-	yes	21.3	deceased	negative	negative	
UPN20	F	46,XX,t(4;12)(q1?1;p1?2)[13]/46,XX[4]	negative	65	int	3.08	DA1, DA2	no	-	no	2.7	deceased	negative	negative	
UPN21	F	47,XX,+4[22]	trisomy 4	74	fav	9	DA1, DA2, MAMAC	yes	-	yes	6	deceased	trisomy 4 plus LOH affecting most of the long arm, including *TET2* (copy number > 2)	negative	
UPN22	F	47,XX,+4[8]/48,XX,+4,+4[7]/48,XX,+4,+8[4]/50,XX,+4,+8,+13,+19[1]	trisomy 4	68	fav	30.8	DA1	no	-	no	0.8	deceased	trisomy 4	negative	
UPN23	M	53,XY,+4,+6,+8,+9,add(17)(q25),+18,+21,+21[26]/46,XY[2]	trisomy 4	18	adv	5.3	MAV	yes	post-CR	yes	7.8	deceased	trisomy 4	negative	
UPN24	F	49,XX,+4,+8,t(10;11)(p13;q14),+12[10]	trisomy 4	53	adv	171	MAV	no	-	no	0.8	deceased	trisomy 4	negative	
UPN25	M	48,XY,+4,-6,+8,+mar[9]/46,XY[21]	trisomy 4	63	adv	14.5	MAV, MAMAC	yes	-	yes	32.9	deceased	trisomy 4 plus LOH affecting most of the long arm, including *TET2* (copy number > 2)	c.4133G>A, p.Cys1378Tyr	biallelic
UPN26	F	47,XX,+4,t(8;21)(q22;q22)[9]/46,XX,t(8;21)(q22;q22)[18]	trisomy 4	52	fav	34.8	MAV, MAMAC	yes	-	yes	17.7	deceased	negative	negative	
UPN27	F	47,XX,+4[18]/46,XX[2]	trisomy 4	64	fav	1.2	DA1, DA2	yes	salvage	yes	22.1	deceased	trisomy 4	negative	
UPN28	M	47,XY,+4[30]	trisomy 4	73	int	36.3	DA1	yes	-	no	62	deceased	trisomy 4 with a focal deletion affecting *TET2* (copy number < 2)	negative	monoallelic
UPN29	F	46,XX,t(9;11)(p21-22;q23)[14]/49,XX,+4,+8,t(9;11)(p21-22;q23),+12[2]	trisomy 4	39	adv	212	DA1	no	-	no	0.1	deceased	negative	negative	
UPN30	F	47,XX,+8[1]/47,der(3)del(3)(p13)ins(3;12)(q21;q13q24.1),del(4)(q2?),+8,der(12)del(12)(q13q24.1)ins(12;4)(q13;?)[21]/47,XX,idem,?add(13)(q34)[2]/46,XX[7]	not discernible	75	adv	4.7	none	no	-	no	23.8	deceased	focal monoallelic deletion	c.3646C>T, p.Arg1216*	biallelic

[a] Risk category according to European LeukaemiaNet (ELN). fav, favourable; int, intermediate; adv, adverse.
[b] Remission-induction treatment regime(s) administered. DA, daunorubicin and Ara-C; MAC, mitoxantrone and Ara-C; MAMAC, amsacrine and Ara-C; MAV, mitoxantrone, Ara-C and VP-16; AZA, 5'-Azacytidine
[c] Patients who received allograft stem cell transplant (ALL-SCT) either after achieving CR (post-CR) or as salvage therapy after relapse (salvage).

Table S8. Clinical characteristics and demographics of AML patients with *TET2* mutation enrolled in the PETHEME FLUGAZA phase 3 clinical trial.

Patient ID	Gender	Age at diagnosis	ECOG score[a]	ELN risk[b]	Treatment[c]	CR	OS (days)	Status (at last follow-up)	Cause of death	*TET2* loss of function mutation status	*TP53* mutation
UPN01 (index case)	M	76	2	fav	DA, AZA	Yes	850	deceased	Progression	biallelic	wild-type
UPN31	M	81	3	adv	AZA	Yes	767	deceased	Progression	biallelic	wild-type
UPN32	M	67	2	fav	AZA	Yes	588	deceased	Progression	monoallelic	wild-type
UPN33	M	70	3	adv	AZA	Yes	579	deceased	Progression	biallelic	c.641A>G, p.His214Arg
UPN34	M	72	1	adv	AZA	No	523	deceased	Progression	monoallelic	wild-type
UPN35	M	77	0	adv	AZA	Yes	517	deceased	Progression	monoallelic	wild-type
UPN36	M	83	2	adv	AZA	No	507	deceased	Progression	monoallelic	wild-type
UPN37	F	73	0	int	AZA	No	470	deceased	Progression	monoallelic	wild-type
UPN38	M	73	0	int	AZA	Yes	435	deceased	Cardiovascular disease	monoallelic	wild-type
UPN39	F	83	3	adv	AZA	Yes	373	deceased	Progression	monoallelic	wild-type
UPN40	M	66	1	adv	AZA	Yes	356	deceased	Progression	monoallelic	wild-type
UPN41	M	77	1	fav	AZA	Yes	344	deceased	Progression	monoallelic	wild-type
UPN42	M	70	2	fav	AZA	No	183	deceased	Progression	monoallelic	wild-type
UPN43	F	86	2	int	AZA	No	125	deceased	Infection	monoallelic	wild-type
UPN44	M	78	0	adv	AZA	No	114	alive	-	monoallelic	wild-type
UPN45	M	71	1	adv	AZA	No	106	deceased	Progression	monoallelic	c.761T>A, p.Ile254Asn
UPN46	F	79	1	adv	AZA	No	82	deceased	Cardiovascular disease	monoallelic	c.338T>C, p.Phe113Ser
UPN47	M	79	3	adv	AZA	No	62	deceased	Progression	biallelic	wild-type
UPN48	M	71	1	adv	AZA	NA	56	deceased	Infection	monoallelic	wild-type
UPN49	M	73	2	adv	AZA	No	52	deceased	Hemorrhagic complication	monoallelic	c.920-2A>G, p.?
UPN50	F	87	3	adv	AZA	No	26	deceased	Progression	monoallelic	wild-type
UPN51	M	70	1	int	FLUGA	Yes	1308	alive	-	monoallelic	wild-type
UPN52	M	71	0	int	FLUGA	No	1023	deceased	Progression	monoallelic	wild-type
UPN53	M	70	0	int	FLUGA	No	862	deceased	Progression	monoallelic	wild-type
UPN54	M	77	1	adv	FLUGA	No	620	deceased	Lung cancer	monoallelic	wild-type
UPN55	F	73	2	int	FLUGA	Yes	551	deceased	Progression	monoallelic	wild-type
UPN56	M	76	1	fav	FLUGA	Yes	473	deceased	Hemorrhagic complication	monoallelic	wild-type

UPN57	M	69	1	fav	FLUGA	Yes	420	deceased	Progression	monoallelic	wild-type
UPN58	F	76	0	adv	FLUGA	Yes	388	deceased	Progression	monoallelic	wild-type
UPN59	M	80	0	adv	FLUGA	Yes	362	deceased	Possible stroke	monoallelic	wild-type
UPN60	F	80	1	adv	FLUGA	Yes	354	deceased	Progression	monoallelic	wild-type
UPN61	F	67	1	int	FLUGA	Yes	267	deceased	Progression	monoallelic	wild-type
UPN62	F	73	1	adv	FLUGA	Yes	220	deceased	Progression	monoallelic	wild-type
UPN63	M	72	0	fav	FLUGA	No	203	deceased	Progression	monoallelic	wild-type
UPN64	F	65	1	int	FLUGA	No	159	deceased	Progression	monoallelic	wild-type
UPN65	M	80	1	adv	FLUGA	No	155	deceased	Progression	monoallelic	c.740A>T, p.Asn247Ile
UPN66	F	70	1	int	FLUGA	No	141	deceased	Progression	monoallelic	wild-type
UPN67	F	82	1	adv	FLUGA	No	126	deceased	Progression	monoallelic	c.920-2A>G, p.?
UPN68	M	72	0	adv	FLUGA	No	111	deceased	Progression	biallelic	wild-type
UPN69	F	67	2	adv	FLUGA	No	100	deceased	Progression	monoallelic	wild-type
UPN70	M	70	2	adv	FLUGA	No	94	deceased	Infection	monoallelic	wild-type
UPN71	M	76	0	int	FLUGA	No	68	deceased	Progression	monoallelic	wild-type
UPN72	M	81	0	int	FLUGA	NA	53	deceased	Myocardial infarction	monoallelic	wild-type
UPN73	F	71	2	int	FLUGA	No	45	deceased	Progression	biallelic	wild-type
UPN74	F	76	1	adv	FLUGA	No	40	deceased	Infection	monoallelic	c.568C>A, p.Pro190Thr
UPN75	F	82	1	adv	FLUGA	Yes	37	deceased	Infection	monoallelic	c.818G>A, p.Arg273His; c.997delC, p.Arg333fs
UPN76	M	81	1	adv	FLUGA	NA	31	deceased	Infection	monoallelic	wild-type
UPN77	F	71	0	fav	FLUGA	NA	19	deceased	Hemorrhagic complication	monoallelic	wild-type
UPN78	M	71	1	int	FLUGA	NA	17	deceased	Hemorrhagic complication	biallelic	wild-type
UPN79	F	80	1	fav	FLUGA	NA	9	deceased	Progression	monoallelic	wild-type
UPN80	M	82	1	adv	FLUGA	No	3	deceased	Tumor lysis syndrome	monoallelic	c.818G>A, p.Arg273His

[a] Performance status according to European Cooperative Oncology Group (ECOG).
[b] Risk category according to European LeukaemiaNet (ELN). fav, favourable; int, intermediate; adv, adverse.
[c] Remission-induction treatment regime(s) administered. DA, daunorubicin and Ara-C; AZA, 5'-Azacytidine; FLUGA, low-dose Ara-C and fludarabine

Table S9. Primer sequences and PCR reaction conditions for Sanger sequencing of genes of interest.

Amplicon	Primer sequences[a]	PCR reaction conditions[b]
TET2 exon 3	F 5'-GCTTTCAAGAACAGGAGCAGA-3' *<br>R 5'-CAGGCATGTGGCTTGCATC-3'	35 cycles:<br>D 95°C 25s<br>A 60°C 35s<br>E 72°C 45s
TET2 exon 6	F 5’-TGCAAGTGACCCTTGTTTTG-3’ *<br>R 5’-TACCGAGACGCTGAGGAAAT-3’	36 cycles:<br>D 95°C 25s<br>A 55°C 35s<br>E 72°C 65s
NPM1 exon 11	F 5’-AACTCTCTGGTGGTAGAATGAAA-3’<br>R 5’-TGAGAACTTTCCCTACCGTGT-3’ *	36 cycles:<br>D 95°C 25s<br>A 55°C 35s<br>E 72°C 65s
FLT3 exon 14	F 5’-ACAGGGACATTGCCTGATTGT-3’<br>R 5’-GGTTGACACCCCAATCCACT-3’ *	35 cycles:<br>D 95°C 25s<br>A 60°C 35s<br>E 72°C 45s

[a] F, forward; R, reverse.
[b] initial denaturation was performed at 95°C for 2 min and the last cycle was followed by a final extension step at 72°C for 5 min in all cases. D, denaturation; A, annealing; E, extension.
* indicates primer used for Sanger sequencing.
